# Integrated multiomic analysis reveals disulfidptosis subtypes in glioblastoma: implications for immunotherapy, targeted therapy, and chemotherapy

**DOI:** 10.3389/fimmu.2024.1362543

**Published:** 2024-02-26

**Authors:** Xue Yang, Zehao Cai, Ce Wang, Chenggang Jiang, Jianguang Li, Feng Chen, Wenbin Li

**Affiliations:** ^1^ Department of Neuro-oncology Cancer Center, Beijing Tiantan Hospital, Capital Medical University, Beijing, China; ^2^ Department of Neurosurgery, Beijing Tiantan Hospital, Capital Medical University, Beijing, China; ^3^ Department of Neurosurgery, Aerospace Center Hospital, Beijing, China

**Keywords:** disulfidptosis, integrated multiomic analysis, immunotherapy, glioblastoma, machine learning

## Abstract

**Introduction:**

Glioblastoma (GBM) presents significant challenges due to its malignancy and limited treatment options. Precision treatment requires subtyping patients based on prognosis. Disulfidptosis, a novel cell death mechanism, is linked to aberrant glucose metabolism and disulfide stress, particularly in tumors expressing high levels of SLC7A11. The exploration of disulfidptosis may provide a new perspective for precise diagnosis and treatment of glioblastoma.

**Methods:**

Transcriptome sequencing was conducted on samples from GBM patients treated at Tiantan Hospital (January 2022 - December 2023). Data from CGGA and TCGA databases were collected. Consensus clustering based on disulfidptosis features categorized GBM patients into two subtypes (DRGclusters). Tumor immune microenvironment, response to immunotherapy, and drug sensitivity were analyzed. An 8-gene disulfidptosis-based subtype predictor was developed using LASSO machine learning algorithm and validated on CGGA dataset.

**Results:**

Patients in DRGcluster A exhibited improved overall survival (OS) compared to DRGcluster B. DRGcluster subtypes showed differences in tumor immune microenvironment and response to immunotherapy. The predictor effectively stratified patients into high and low-risk groups. Significant differences in IC50 values for chemotherapy and targeted therapy were observed between risk groups.

**Discussion:**

Disulfidptosis-based classification offers promise as a prognostic predictor for GBM. It provides insights into tumor immune microenvironment and response to therapy. The predictor aids in patient stratification and personalized treatment selection, potentially improving outcomes for GBM patients.

## Introduction

Glioblastoma multiforme (GBM), the most common primary malignant tumor of the central nervous system in adults, poses significant therapeutic challenges. Current standard treatments yield limited efficacy, with a 5-year overall survival rate of only 6.8% (1). First-line therapy typically involves maximal safe tumor resection, followed by radiotherapy (RT) and concurrent temozolomide (TMZ) chemotherapy. While combination therapy extends median overall survival (OS) compared to radiotherapy alone, with durations of 14.6 months and 12.1 months, respectively, the outcomes remain constrained. Limited options exist for recurrent GBM, including second-line surgery, radiotherapy, alkylating agent chemotherapy, and bevacizumab treatment. Unfortunately, from the onset of progression or recurrence, the median OS is merely 6 to 9 months (2). Urgent exploration of novel therapeutic strategies for recurrent GBM is imperative.

Previous studies have underscored the profound association between the prognosis of glioblastoma patients and the regulatory patterns of cell death, such as apoptosis, ferroptosis (3), and copper-dependent cell death (4). In this context, disulfidptosis has emerged as a recently revealed mechanism, inducing cell death through the accumulation of disulfide bonds leading to the collapse of the actin cytoskeleton. Proteins crucially implicated in disulfidptosis, such as SLC7A11 and NCKAP1 (5), are closely linked to the occurrence and progression of glioblastoma. Disulfide stress triggers disulfide cross-linking within cytoskeletal proteins, initiating cell contraction, membrane instability, and subsequent cell death. Insufficient glucose uptake and excessive cysteine intake both induce disulfidptosis. This form of cell death, subject to modulation by drugs targeting proteins like SLC7A11 and NCKAP1, holds potential therapeutic value (6).

Immunotherapy, as a novel approach harnessing the patient’s immune system to combat tumors, has shown significant progress in various cancers (7). Although breakthroughs in GBM treatment remain elusive, recurrent GBM following prior radiotherapy and chemotherapy tends to exhibit higher mutational loads and immunogenicity (8), offering a glimmer of hope for immunotherapy. Future research directions encompass a deeper understanding of GBM biology, the immune microenvironment, and the development of innovative treatment combinations (8).

There is a dearth of research exploring the correlation between disulfidptosis and the immune microenvironment in GBM, along with its effects on functional outcomes and responses to immunotherapy, targeted therapy, and chemotherapy drugs. We collected samples from 26 GBM patients undergoing surgical treatment at the Tiantan Hospital, Capital Medical University from January 2022 to December 2023, conducted transcriptome sequencing, and integrated data from the CGGA and TCGA databases. Utilizing disulfidptosis characteristics, we employed consensus clustering to classify GBM patients into two subtypes (DRGcluster A and B) with distinct survival outcomes, functional annotations, and clinical features. Through comprehensive analysis, we revealed disparities in genomic variations, tumor microenvironments, and immune genomic patterns between the two subtypes, concurrently identifying distinct benefits of chemotherapy, targeted therapy, and immunotherapy. Leveraging the LASSO machine learning algorithm, we developed a disulfidptosis high-low-risk subtype predictor comprising 8 genes, subsequently validated for survival in GBM cohorts from CGGA and TCGA. The pRRophetic algorithm facilitated subtype analysis, offering a potential means to identify patients more likely to respond positively to chemotherapy, targeted therapy, and immunotherapy. The objective of our study is to enhance personalized survival prognostication through an innovative disulfidptosis molecular classification, thus offering enhanced therapeutic alternatives for physicians and GBM patients.

## Materials and methods

### Patient cohorts and multiomic data collection

Gene expression data, clinical information, and follow-up data for glioblastoma (GBM) patients were obtained from The Cancer Genome Atlas (TCGA) via the UCSC Xena platform (https://xenabrowser.net/). Concurrently, RNA sequencing (RNA-Seq) data from GBM patients generated using the Illumina HiSeq platform were retrieved from the Chinese Glioma Genome Atlas (CGGA) database (http://www.cgga.org.cn). After excluding samples with incomplete clinical information and a total survival period of less than 3.5 months, we ultimately enrolled 135 GBM patients from TCGA and 314 from the CGGA database, ensuring the integrity of scientific data. Demographic and follow-up data for GBM patients are presented in **Table 1**. Furthermore, somatic mutation data of 390 GBM patients were analyzed and visualized using the maftools and GenVisR packages in R. Tumor Mutational Burden (TMB), a potential biomarker for immunotherapy response, was defined as the total number of nonsynonymous mutations per megabase in the coding region. Copy Number Alteration (CNA) data of 628 GBM patients were also obtained from the TCGA dataset, with significant amplifications or deletions across the genome identified using GISTIC 2.0. Circos plots were generated using the RCircos package in R to visualize chromosomal gains and losses. CNA burden was defined as the total number of genes with copy number changes in each sample.

**Table 1:** Demographics and clinicopathological features of GBM patients in the CGGA, TCGA and Tiantan cohort.

Variables	CGGA cohort(n=314)			TCGA cohort(n=135)			Tiantan cohort(n=26)		
	DRGcluster B N=178	DRGcluster A N=136	p.overall	DRGcluster B	DRGcluster A	p.overall	DRGcluster B	DRGcluster A	p.overall
				N=61	N=74		N=11	N=15	
Age:			0.515			1			0.683
~64	160 (89.9%)	126 (92.6%)		40 (65.6%)	49 (66.2%)		7 (63.6%)	11 (73.3%)	
65~	18 (10.1%)	10 (7.35%)		21 (34.4%)	25 (33.8%)		4 (36.4%)	4 (26.7%)	
Gender:			0.031			1			1
Female	60 (33.7%)	63 (46.3%)		20 (32.8%)	24 (32.4%)		4 (36.4%)	5 (33.3%)	
Male	118 (66.3%)	73 (53.7%)		41 (67.2%)	50 (67.6%)		7 (63.6%)	10 (66.7%)	
Chemotherapy:			0.62			1			NA
YES	144 (80.9%)	113 (83.1%)		45 (73.8%)	51 (68.9%)		11 (100%)	15 (100%)	
NO	27 (15.2%)	17 (12.5%)		14 (23.0%)	17 (23.0%)		0	0	
unknow	7 (3.93%)	6 (4.41%)		2 (3.28%)	6 (8.11%)		0	0	
Radiotherapy:			0.152			0.567			NA
YES	147 (82.6%)	105 (77.2%)		50 (82.0%)	61 (82.4%)		11 (100%)	15 (100%)	
NO	21 (11.8%)	25 (18.4%)		9 (14.8%)	7 (9.46%)		0	0	
unknow	10 (5.62%)	6 (4.41%)		2 (3.28%)	6 (8.11%)		0	0	
X1p19q:			<0.001			NA			NA
Non-Codel	177 (99.4%)	115 (84.6%)		59 (96.7%)	70 (94.6%)		8 (72.7%)	9 (60.0%)	
Codel	1 (0.56%)	18 (13.2%)		0	0		0	0	
unknow	0 (0.00%)	3 (2.21%)		2 (3.28%)	4 (5.41%)		3 (27.3%)	6 (40.0%)	
MGMT:			<0.01			0.443			1
Unmethylated	79 (49.4%)	44 (34.9%)		22 (36.1%)	38 (51.4%)		4 (36.4%)	6 (40.0%)	
Methylated	63 (39.4%)	67 (53.2%)		22 (36.1%)	26 (35.1%)		4 (36.4%)	4 (26.7%)	
unknow	18 (11.2%)	15 (11.9%)		17 (27.9%)	10 (13.5%)		3 (27.3%)	5 (33.3%)	
IDH:			<0.001			0.025			1
Wildtype	157 (88.2%)	74 (54.4%)		58 (95.1%)	60 (81.1%)		6 (54.5%)	8 (53.3%)	
Mutant	16 (8.99%)	57 (41.9%)		1 (1.64%)	10 (13.5%)		2 (18.2%)	2 (13.3%)	
unknow	5 (2.81%)	5 (3.68%)		2 (3.28%)	4 (5.41%)		3 (27.3%)	5 (33.3%)	
Chr_7_gain_Chr_10_loss:			NA			0.662			0.335
Gain chr 7 & loss chr 10	NA	NA		41 (67.2%)	45 (60.8%)		5 (45.5%)	4 (26.7%)	
No combined CNA	NA	NA		18 (29.5%)	25 (33.8%)		2 (18.2%)	6 (40.0%)	
unknow	NA	NA		2 (3.28%)	4 (5.41%)		4 (36.4%)	5 (33.3%)	

GBM, glioblastoma; NA, not available;MGMT,O6-methylguanine-DNA methyltransferase;IDH,isocitrate dehydrogenase.

### Sample collection from Tiantan Hospital, Capital Medical University

From January 2022 to December 2023, we collected 26 fresh frozen GBM specimens with complete clinical information. We excluded patients with incomplete clinical data and those with a survival time of less than 3.5 months. Diagnoses were confirmed through histopathological examination, and clinical information was obtained via electronic medical records or telephone follow-up. Demographic and follow-up data for these 26 GBM patients are shown in **Table 1**. The study was approved by the Ethics Committee of Tiantan Hospital, Capital Medical University (Ethical Code: YW2022-025), and adhered to the ethical standards of the Helsinki Declaration and its later amendments. Informed consent was obtained from all participants through agreements with TCGA, CGGA member institutions, and Tiantan Hospital.

### Transcriptomic next-generation sequencing of Tiantan samples

We performed RNA sequencing on Tiantan Hospital samples using the Illumina HiSeq platform. RNA integrity and quantity were assessed using the Agilent 2100 Bioanalyzer. Library construction involved mRNA enrichment, fragmentation, cDNA synthesis, end-repair, A-tailing, and sequencing adapter ligation. Libraries were size-selected, PCR amplified, and quantified. Sequencing reads were aligned to the hg38 reference genome using STAR, and gene expression levels were quantified using featureCounts and FPKM.

### Tumor immune microenvironment (TIME) patterns and immunogenomic features in glioblastoma (GBM)

Utilizing expression data (ESTIMATE), we assessed GBM samples to evaluate the tumor microenvironment and predict tumor purity as well as the abundance of infiltrating stromal and immune cells. ESTIMATE generates four scores: Immune Score (reflecting the abundance of immune cells), Stromal Score (reflecting the abundance of stromal cells), ESTIMATE Score (reflecting non-tumor constituents), and Tumor Purity. Additionally, we applied the CIBERSORT deconvolution algorithm, based on linear support vector regression, to quantify the composition of 22 types of tumor-infiltrating immune cells (TIICs) based on the gene expression profiles of GBM samples. Single-sample Gene Set Enrichment Analysis (ssGSEA) was utilized to quantify the enrichment levels of these immune gene sets. Subsequently, unsupervised hierarchical clustering based on ssGSEA scores of 29 immune markers categorized GBM patients into different clusters, i.e., immune subtypes.

### Differential analysis between DRGcluster A and B groups

To explore a novel molecular classification of GBM patients based on Differential Expressed Genes (DEGs), we employed unsupervised consensus clustering using the k-means machine learning algorithm. We selected 32 genes related to disulfidptosis, identified from published literature and GeneCards (e.g., FLNA, FLNB, MYH9, TLN1, ACTB, MYL6, MYH10, CAPZB, DSTN, IQGAP1, ACTN4, etc.). The consensus clustering was performed with 1000 iterations, each involving 80% data sampling. The optimal number of clusters was comprehensively determined by the relative change in the area of the Cumulative Distribution Function (CDF) curve, the Proportion of Ambiguous Clustering (PAC) algorithm, and consensus heatmap. We compared the clinicopathological parameters within different clusters to further explore the association between disulfidptosis subtypes and clinical characteristics of GBM patients. Based on consensus analysis, GBM patients were classified into DRGcluster A and B groups. The overall survival (OS) of patients in DRGcluster A and B was assessed using Kaplan-Meier survival analysis, and survival differences were evaluated using a two-sided log-rank test.

### Analysis of differential gene expression between DRGcluster A and B groups using limma package in R

We employed the limma package in R to screen for Differential Expressed Genes (DEGs) between DRGcluster A and B groups. P-values were adjusted using the default Benjamini-Hochberg False Discovery Rate (FDR) method. DEGs with FDR < 0.01 and |fold change (FC)| > 1.5 were considered significant. Subsequently, functional annotation and pathway enrichment analyses of DEGs were performed using the WebGestaltR package in R, including Gene Ontology (GO) and Kyoto Encyclopedia of Genes and Genomes (KEGG) pathway analyses. Results with FDR < 0.05 were considered statistically significant.

### Gene set variation analysis (GSVA)

We assessed the most significantly enriched molecular pathways in disulfidptosis subtypes using the GSVA package in R. Differential analysis of KEGG pathway enrichment scores between the two subtypes was conducted using the limma package in R. KEGG pathways with |fold change (FC)| > 1.5 and FDR < 0.05 were considered the most significantly enriched molecular pathways between the two disulfidptosis subtypes.

### Prediction of response to chemotherapy, targeted therapy, and immunotherapy

We predicted the response of GBM samples to chemotherapy and targeted therapy using the pRRophetic package. The response was determined by the half-maximal inhibitory concentration (IC50) for each GBM sample. After integrating the expression profiles of cell lines (training set) and GBM samples (test set), regression analysis estimated the IC50 for common chemotherapeutic and targeted drugs in GBM patients. Predictive accuracy was evaluated using 10-fold cross-validation. Considering the potential limitations of TIDE in predicting treatment responses in cancer types other than melanoma and non-small cell lung cancer, we analyzed differential expression of immune molecular markers in different DRGcluster groups and high/low-risk groups to predict responses to relevant immunotherapies. Results with p-value < 0.05 were considered statistically significant.

### Machine learning construction and validation of a High/Low-Risk Disulfidptosis Subtype Predictor

Gene sets associated with glioblastoma occurrence from the WGCNA model and those showing significant differences between DRGcluster A and B patients were selected and visualized using Venn diagrams. A total of 314 CGGA GBM patients were used as a training set, and 135 TCGA GBM patients as a test set. In the training set, the most relevant features associated with the groups were selected using LASSO regression analysis in the glmnet package in R, constructing a predictor named “High/Low-Risk Disulfidptosis Predictor”. The performance of the predictor was studied through standard and calibration plots. Finally, survival analysis in the test set validated the predictive functionality of the High/Low-Risk Disulfidptosis Predictor.

### Quality control of scRNA-seq data

We collected a total of 42 samples from 16 GBM patients from GSE182109 for analysis. Python package Scanpy (V1.8.2) (9) was used to merge the raw count matrix of each sample and subsequently conduct a quality control analysis. For gene filtering, genes that were expressed in less than 3 cells were removed. For cell filtering, cells were selected with the following principles: (1) the number of expressed genes was from 200 to 10000, (2) the mitochondrial RNA content was lower than 20%, and (3) the total counts of each cell ranged from 100 to 50,000. Then, Scrublet (https://github.com/swolock/scrublet) (10) was used to detect potential doublets in each sample (doublet_score<0.3). Finally, a total of 227,584 single-cell transcriptomes were retained after quality control.

### Dimensionality reduction and clustering

Gene expression was normalized using function sc.pp.normalize_total(adata, target_sum=1e4) and log1p shifted. Highly variable genes (HVGs) were generated with the parameter (min_mean=0.0125, max_mean=3, min_disp=0.5) and 2414 HVGs were selected. Principal component analysis (PCA) on the gene expression matrix and used the first 50 principal components (PCs) for UMAP. The batch effect removal used BBKNN (https://github.com/Teichlab/bbknn) (11).

### Gene signature score analysis

In this study, we used the function score_genes in Scanpy with default parameters to quantify the activity of gene sets in macrophages and microglia. The gene sets for evaluating macrophages and microglia were gained from Zhang et al. (12)and Lawson el al (13).

### Statistical analysis

Inter-group statistical comparisons for continuous data were performed using independent Student’s t-tests, χ2 tests for categorical data, and Mann-Whitney U tests for comparing categorical and non-normally distributed variables. The Kruskal-Wallis test was used for multi-group comparisons. Pearson correlation was employed to assess relationships between normally distributed variables, while Spearman correlation was used for non-normally distributed variables. Statistical analyses in this study were conducted using SPSS 27.0, Perl, R 4.3.1, and R 4.1.3. Two-sided p-values < 0.05 were considered statistically significant. The R packages used can be obtained from the website http://bioconductor.org/. The overall workflow of this study is shown in **Supplementary Figure 1**.

## Results

### Association between disulfidptosis classification and clinical features

**Figure 1:**
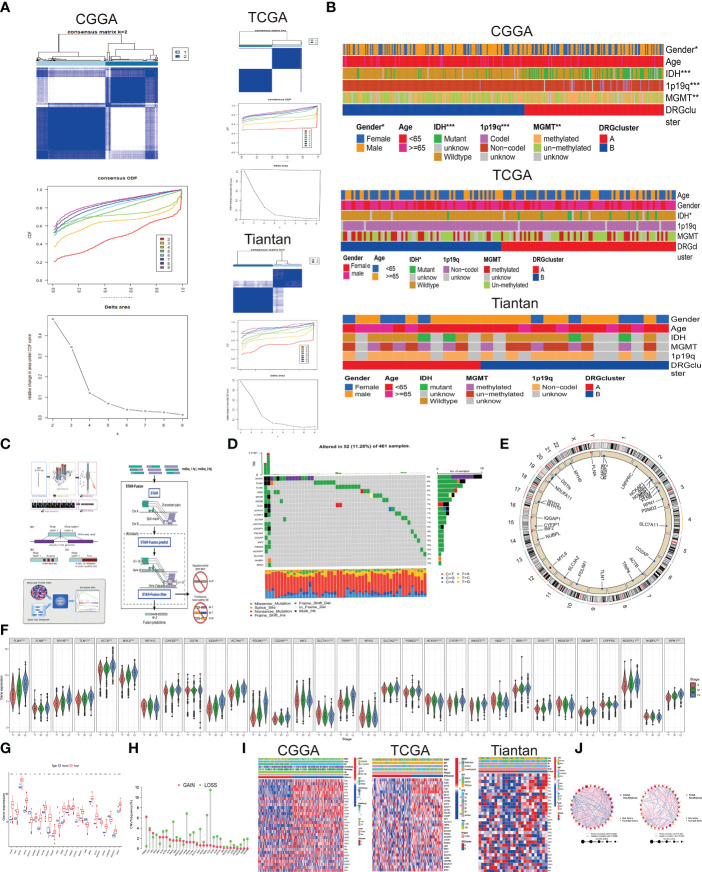
Association between disulfidptosis classification and Clinical Features **(A)** Based on 32 genes associated with disulfidptosis, glioblastoma multiforme (GBM) patients were stratified into two subgroups (k = 2) through consensus clustering analysis. Left panel displays data from CGGA database, top right panel from TCGA database, and bottom right panel from Tiantan database. **(B)** Clinical characteristics differentiating patients in DRG cluster A and B across the three databases are presented through a heatmap analysis. **(C)** The transcriptome sequencing workflow for Tiantan patient samples is provided by Beijing Nuohe Zhiyuan Biotechnology Co., Ltd. **(D)** Frequency and types of mutations in disulfidptosis-associated genes in TCGA database. **(E)** Circos plot illustrating the genomic distribution of disulfidptosis genes on different chromosomes. **(F)** Violin plots depicting the expression differences of disulfidptosis genes in patients classified under WHO II, III, and IV categories. (Statistical significance denoted by p-values: *p<0.05, **p<0.01, ***p<0.001). **(G)** Differential expression of disulfidptosis genes between GBM and normal brain tissues in TCGA database. **(H)** Frequency and types of copy number variations (CNV) mutations in disulfidptosis genes. **(I)** Heatmap displaying the relationship between expression of disulfidptosis genes, patient grouping, and clinical characteristics across CGGA, TCGA, and Tiantan databases.J. Prognostic network diagram elucidating the association between DRGclusters and the prognosis of GBM. (Risk factors denoted by high-risk genes; favorable factors indicated by low-risk genes; The size of the node corresponds to the P value).

Utilizing unsupervised consensus clustering, we explored a novel molecular classification of glioblastoma multiforme (GBM) patients based on differential expression patterns of 32 disulfidptosis -associated genes. The optimal clustering number was determined as two (k=2) based on relative changes in the cumulative distribution function (CDF) area, PAC algorithm, and consensus heatmap, exhibiting high consistency across TCGA, CGGA, and Tiantan samples (**Figure 1A**). Subsequently, all GBM patients were categorized into two groups, DRGcluster A and B. We further investigated the association between different groups and clinical features (**Figure 1B** and **Table 1**). As shown in **Figure 1B**, in the CGGA database, the proportion of males was higher in the DRGcluster A group, indicating a significant gender difference (p=0.031). In the DRGcluster B group, a higher tendency toward IDH mutation (P<0.001), 1p19q chromosomal codeletion (P<0.001), and MGMT promoter methylation (P=0.01) was observed, demonstrating significant group differences (**Figure 1B**). In the TCGA GBM cohort, we also found a significant group difference in IDH mutation, with the DRGcluster B group showing a higher tendency toward IDH mutation. Additionally, we observed that mesenchymal patients were more likely to be in the DRGcluster A group, while neural tumor patients were more likely to be in the DRGcluster B group. We summarized the data from Tiantan as well, where no significant clinical feature differences were observed between DRGcluster A and B groups. Notably, the DRGcluster B group showed a higher occurrence of chromosome 7 amplification and chromosome 10 deletion, consistent with the data from the TCGA database. Moreover, we presented the process of transcriptome sequencing for the 26 clinical samples collected in our study, highlighting the similarity in data processing procedures with TCGA and CGGA datasets, ensuring data uniformity (**Figure 1C**).

Combining copy number alteration (CNA) analysis and somatic mutation analysis, we conducted an in-depth analysis of genes included in the disulfidptosis cell subtypes. The results revealed mutations in genes such as MYH3, FLNA, FLNB, ABI2, MYH10, OXSM, and TLN1D, with MYH exhibiting a 3% somatic mutation rate, predominantly in the form of frame-shift insertions, missense mutations, and multi-hits. Most gene mutations were missense mutations (**Figure 1D**). In **Figures 1E, H**, we presented the CNV frequency statistics for the 32 genes, indicating their chromosomal locations. Genes were located on chromosomes 1, 2, 3, 4, 6, 7, 9, 10, 11, 12, 14, 15, 17, 19, 20, 22, and X, with most genes experiencing deletions as the primary CNV change. Additionally, we used the CGGA database to analyze the expression differences of the 32 disulfidptosis-associated genes in WHO II, III, and IV populations, revealing significant expression differences (p<0.001). Differential analysis of gene expression data from GBM tumor and normal patients in the TCGA database also confirmed these results (**Figures 1F, G**), demonstrating the relevance of the gene set included in the disulfidptosis cell subtype to the occurrence and development of GBM. Subsequently, we used a heatmap to analyze the differential expression of disulfidptosis -associated genes between different DRGcluster groups, showing significant differences and consistent findings across CGGA, TCGA, and Tiantan datasets (**Figure 1I**). We used the corr.test function in the CGGA and TCGA database to calculate the correlation of transposed gene expression Data (**Figure 1J**), with purple nodes indicating risk factors and green nodes indicating favorable factors. IQGAPI, PDLIM1, FLNA, MYH9, and TLN1 were significant risk factors (p<0.001), and no significant differences were observed in favorable factors. Pink lines represented positive correlations, while blue lines represented negative correlations. There was a close correlation (p<0.0001) among the 32 genes, indicating the profound research value of this disulfidptosis gene set.

### The association between DRGclusters and the immune microenvironment pattern

**Figure 2:**
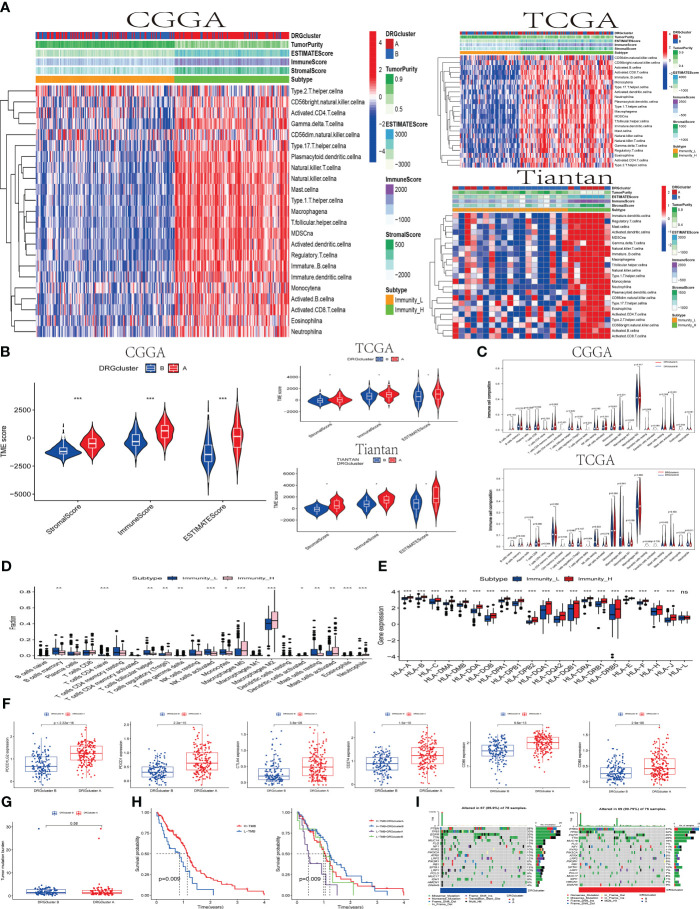
The association between the DRGclusters and the immune microenvironment pattern **(A)** A heatmap was used to illustrate the relationship between DRGclusterA and B subgroups of patients from the CCGA, TCGA, and Tiantan cohorts, and their association with high/low immune groups and immune scores. **(B)** Evaluation of the differences in tumor microenvironment scores between DRGcluster **(A, B)** subgroups in the CCGA, TCGA, and Tiantan cohorts. **(C)** Comparison of immune cell infiltration differences between DRGcluster **(A, B)** subgroups in the CGGA and TCGA cohorts. **(D)** Box plots depicting differences in immune cell infiltration among patients with different immune subtypes. **(E)** Differential expression of HLA family genes in patients stratified by high and low immune groups. **(F)** Scatter plots comparing the expression levels of multiple immune-related genes in different DRGcluster subtypes. **(G)** Differences in tumor mutation burden among patients in different DRGcluster subgroups. **(H)** Survival curve analysis demonstrating the survival probabilities of patients in different immune subtypes and combined DRGcluster subtypes. **(I)** A heatmap illustrating the gene mutation profiles in samples from different DRGcluster subgroups of patients.

Firstly, we conducted a quantitative analysis of the overall immune activity of GBM using the ssGSEA technique, focusing on 29 immune-related gene sets. An unsupervised hierarchical clustering method was utilized to categorize patients from CGGA, TCGA, and Tian Tan databases into different immune subtypes. In this classification, the high-immunity group (Immunity_H) refers to tumors with the highest enrichment scores, classified as “immune-active.” Conversely, the low-immunity group (Immunity_L) includes tumors with the lowest enrichment scores, identified as “immune-inactive.” Notably, the Immunity_H subtype was more prevalent in DRGcluster A, whereas Immunity_L was more common in DRGcluster B. This finding was validated in studies from CGGA, TCGA, and Tian Tan samples (as shown in **Figure 2A**).

Subsequently, the tumor immune environment (temporal) patterns were assessed using ESTIMATE and CIBERSORT algorithms. In DRGcluster A, higher immune, stromal, and ESTIMATE scores (P < 0.001) were observed, suggesting a lower level of immune and stromal cell infiltration in DRGcluster B (as depicted in **Figure 2B**). Moreover, tumors in DRGcluster B exhibited higher purity. Additionally, high-immunity tumors showed significantly higher immune and stromal scores, indicating a richer content of immune and stromal cells in these tumors. Data from TCGA and Tian Tan also demonstrated high consistency. It is worth mentioning that changes in the tumor microenvironment are closely related to the occurrence of disulfidoptosis.

Furthermore, we quantified the abundance of 22 types of immune cells using the CIBERSORT algorithm (refer to **Figures 2C–E**). Analysis from the CGGA database indicated significant associations between different T cell subgroups (including regulatory T cells (Tregs), γδ T cells, CD4 memory resting T cells, activated NK cells, plasma cells, M0 macrophages, and monocytes) in the DRGcluster groups experiencing disulfidoptosis. In the TCGA database, DRGcluster B showed more significant infiltration of M0 macrophages and resting NK cells, consistent with CGGA’s findings. DRGcluster A, on the other hand, exhibited higher infiltration of M1 and M2 macrophages. Although there was no significant difference in M2 macrophage infiltration between the two groups, both CGGA and TCGA data showed more pronounced M2 macrophage infiltration in group A compared to group B.

In **Figures 2D, E**, the CGGA database analysis focused on the proportion differences of various types of immune cells between the Immunity_L and Immunity_H subtypes. Significant differences in the proportions of B cells, plasma cells, various T cells (CD8, CD4), NK cells, and macrophages were observed between these subtypes, highlighting the differing immune cell infiltrations in Immunity_L and Immunity_H. HLA genes play a crucial role in the immune system’s recognition of foreign substances, as their expression levels affect the immune response to cancer cells. The data clearly showed significant differences in the expression levels of various HLA genes, apart from HLA-L, between the Immunity_L and Immunity_H subtypes, revealing the importance of high and low immunity subtypes in the immune response of GBM. In **Figure 2F**, it was evident that the expression of molecules like CD80, CD86, CTLA4, PDCD1, PDCD1LG2, and CD274 was higher in the DRGcluster A group than in the DRGcluster B group, with significant differences in the expression of these checkpoint molecules between the two groups. This suggests that patients in the DRGcluster A might be more suitable for treatment with immune checkpoint inhibitors to more effectively activate the immune response. We conducted data analysis on both the TCGA database and 26 samples from Tian Tan Hospital simultaneously, and found that their trends were roughly similar, with most P-values being statistically significant. This result validates the conclusions we obtained from the analysis of the CGGA database previously (shown in the **Supplementary Figures 2A–F**).

**Figure 2G** showed no significant difference in the tumor mutation burden (TMB) between DRGcluster A and B. Interestingly, when patients were stratified based on high/low TMB and DRGclusters, the Kaplan-Meier survival curves indicated a higher survival rate in the high TMB group, with a p-value of 0.009 (**Figure 2H**). Finally, **Figures 2I** presented the gene variation scenarios between DRGcluster A and B, focusing on mutations, amplifications, and deletions. The presence of various key tumor-driving molecules, including IDH, in these groups highlighted the importance of these immune landscapes and gene variations in deepening our understanding of the impact of disulfidoptosis on the immune microenvironment and tumor development.

### The relationship between DRGcluster A and B groups and GBM survival and molecular pathways

**Figure 3:**
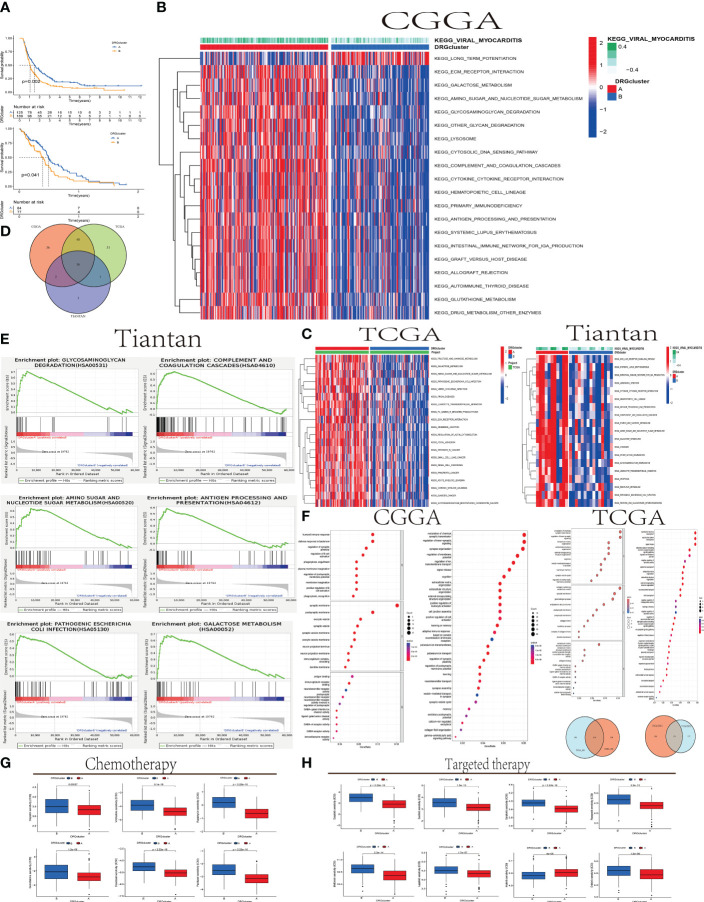
The relationship between DRGcluster **(A, B)** GBM Survival and Molecular Pathways **(A)** Survival analysis plot comparing the survival times of patients in DRGcluster **(A, B)** subgroups from CGGA and TCGA cohorts. **(B, C)** Heatmaps displaying differences in Gene Set Variation Analysis (GSVA) among samples from different DRGcluster subgroups in CGGA, TCGA, and Tiantan cohorts. **(D)** Venn diagrams illustrating common or unique elements in GSVA analysis among different DRGcluster subgroups in CGGA, TCGA, and Tiantan samples. **(E)** GSEA analysis of different DRGcluster subgroups in Tiantan samples. **(F)** Venn diagrams depicting common or unique elements in Gene Ontology (GO) and Kyoto Encyclopedia of Genes and Genomes (KEGG) analyses among different DRGcluster subgroups in CGGA and TCGA samples. **(G, H)** Displaying treatment responses specific to chemotherapy and targeted drugs.

In this study, we applied consensus clustering to classify populations in the CGGA and TCGA databases into DRGcluster A and B groups. Subsequent Kaplan-Meier survival analysis in both databases revealed that the OS (Overall Survival) of the DRGcluster A group was significantly better (CGGA, log-rank P=0.002; TCGA, log-rank P=0.041). This finding corresponds with earlier immune analysis results, indicating that the DRGcluster A group, characterized by higher immune activity, exhibited improved survival (**Figure 3A**). We also presented the correlation analysis of overall survival time among GBM patients with different clinical features and DRGcluster subgroups in **Table 2**.

**Table 2:** Univariate and multivariate cox proportional hazards analysis of clinicopathological variables based on overall survival (OS) in the CGGA cohort, TCGA cohort.

For OS variables	CGGA cohort (n = 314)				TCGA cohort (n =135)			
	Univariate analysis		Multivariate analysis		Univariate analysis		Multivariate analysis	
	P-value	HR (95% CI)	HR (95% CI)	P-value	P-value	HR (95% CI)	P-value	HR (95% CI)
Age	0.51	1.1 (0.77-1.7)	–	–	0.00044	2.3 (1.4-3.6)	0.011	1.9 (1.2-3.2)
Gender	0.66	1.1 (0.82-1.4)	−	−	0.4	0.83 (0.54-1.3)	−	−
Chemotherapy	0.000086	1.9 (1.4-2.7)	2 (1.4-2.8)	0.00011	0.076	1.5 (0.96-2.4)	–	–
Radiotherapy	0.096	1.3 (0.95-1.9)	–	–	0.00081	2.6 (1.5-4.7)	0.00028	3.2 (1.7-6)
IDH mutation status	0.04	0.74 (0.55-0.99)	0.84 (0.6-1.2)	0.31	0.0048	0.23 (0.082-0.64)	0.09	0.34 (0.099-1.2)
MGMT promoter methylation status	0.088	0.79 (0.6-1)	–	–	0.087	0.64 (0.38-1.1)	–	–
DRGcluster subtype	0.0028	0.69 (0.54-0.88)	0.75 (0.56-0.99)	0.044	0.088	0.69 (0.45-1.1)	–	–

OS, overall survival; GBM, glioblastoma; HR, hazard ratio; CI, confidence interval.

In **Figures 3B, C**, we conducted GSVA (Gene Set Variation Analysis) comparisons between DRGcluster A and B in the CGGA, TCGA databases, and data from Tiantan patients. In **Figure 3D**, we merged the GSVA analyses from all three databases, identifying 16 enriched intersecting pathways. All these pathways were positively correlated with the DRGcluster A group and primarily associated with regulation of glucose metabolism, immune stress modulation, apoptosis, and lysosomal function. Enrichment analysis revealed upregulation of genes related to glycosaminoglycan degradation in the DRGcluster A samples, suggesting active glycosaminoglycan metabolism, positively correlated with the development of disulfidptosis. This indicates that cellular metabolic regulation during disulfidptosis may represent an adaptive response of GBM under stress conditions.

Additionally, in the DRGcluster A samples, elevated gene expression levels were observed in pathways such as KEGG_GALACTOSE_METABOLISM, KEGG_OTHER_GLYCAN_DEGRADATION, and KEGG_AMINO_SUGAR_AND_NUCLEOTIDE_SUGAR_METABOLISM, all closely related to abnormal sugar metabolism. These pathway enrichments, positively correlated with DRGcluster A, suggest significant adjustments and changes in cellular metabolic pathways during disulfidptosis. In the DRGcluster A group, pathways including KEGG_COMPLEMENT_AND_COAGULATION_CASCADES, KEGG_CYTOKINE_CYTOKINE_RECEPTOR_INTERACTION, KEGG_LEUKOCYTE_TRANSENDOTHELIAL_MIGRATION, and KEGG_ANTIGEN_PROCESSING_AND_PRESENTATION were upregulated, indicating higher immune activity in these patients. This may reflect an interaction between disulfidptosis and the immune system, leading to immune activation. Genes related to KEGG_LYSOSOME were also positively correlated with DRGcluster A, underlining the lysosome’s key role in cellular waste management, particularly under conditions of disulfidptosis.

In **Figure 3E**, GSEA (Gene Set Enrichment Analysis) was performed on Tiantan data, selecting six pathways for demonstration. This analysis showed high consistency with the GSVA results. For instance, Glycosaminoglycan Degradation (hsa00531) and Galactose Metabolism (hsa00052) pathways had the highest Enrichment Scores (ES) around 0.7, indicating significant upregulation of some genes in these pathways in the DRGcluster A group. The Amino Sugar and Nucleotide Sugar Metabolism (hsa00520) pathway had an ES peak near 0.6. Complement and Coagulation Cascades, and Antigen Processing and Presentation (hsa04612) pathways also showed gene upregulation, with the highest ES points around 0.5. The Pathogenic Escherichia coli Infection (hsa05130) pathway had an ES below 0.5, suggesting a moderate upregulation of genes in this pathway in the DRGcluster A group, though not significantly prominent.

In our study, we utilized the TCGA and CGGA databases to conduct GO and KEGG pathway analyses. The GO analysis revealed 114 pathways that were significantly enriched in both datasets, indicating a substantial consistency in functional annotation between TCGA and CGGA. Similarly, the KEGG analysis identified 170 common pathways, further supporting the concordance in functional annotations between the two datasets.In the GO analysis, certain biological processes such as immune response, cell signaling, and nervous system development exhibited a higher proportion of genes and significance. This suggests that these biological processes may play a crucial role in the process of disulfidptosis. Regarding cellular components and molecular functions enrichment, disulfidptosis was associated with cellular structures such as post-synaptic membrane, synaptic vesicle membrane, neurite membrane, and immune globulin complexes in circulation. Molecular functions related to immune globulin receptor binding, GABA-gated chloride ion channel activity, GABA receptor activity, antigen binding, and neurotransmitter-gated ion channel activity involved in the regulation of post-synaptic membrane potential were highlighted.The significance of GABA receptor activity implies that GABAergic signaling may be a key aspect in neuroconduction-related studies. The notable significance of antigen binding suggests that antigen binding may be a crucial point in studies related to immune regulation (**Figure 3F**).

In **Figures 3G, H**, we employed the pRRophetic package to analyze the TCGA database and the Cancer Therapeutics Response Portal (CTRP) drug library. pRRophetic utilized these data to predict the sensitivity of DRGclusterA and B group patients to specific drugs based on gene expression patterns. The results demonstrated significant differences in sensitivity to various drugs, including most chemotherapy drugs such as cisplatin, gemcitabine, and vinblastine, as well as targeted drugs like lapatinib, dasatinib, and sunitinib (p < 0.001). Group A exhibited lower IC50 values, suggesting higher sensitivity to these drugs compared to Group B. Conversely, the targeted drug imatinib showed higher IC50 values in DRGclusterA (p < 0.001), indicating lower sensitivity in Group A compared to Group B.

### The establishment of a disulfidptosis high and low-risk model and the prediction of immunological landscape and survival risk

**Figure 4:**
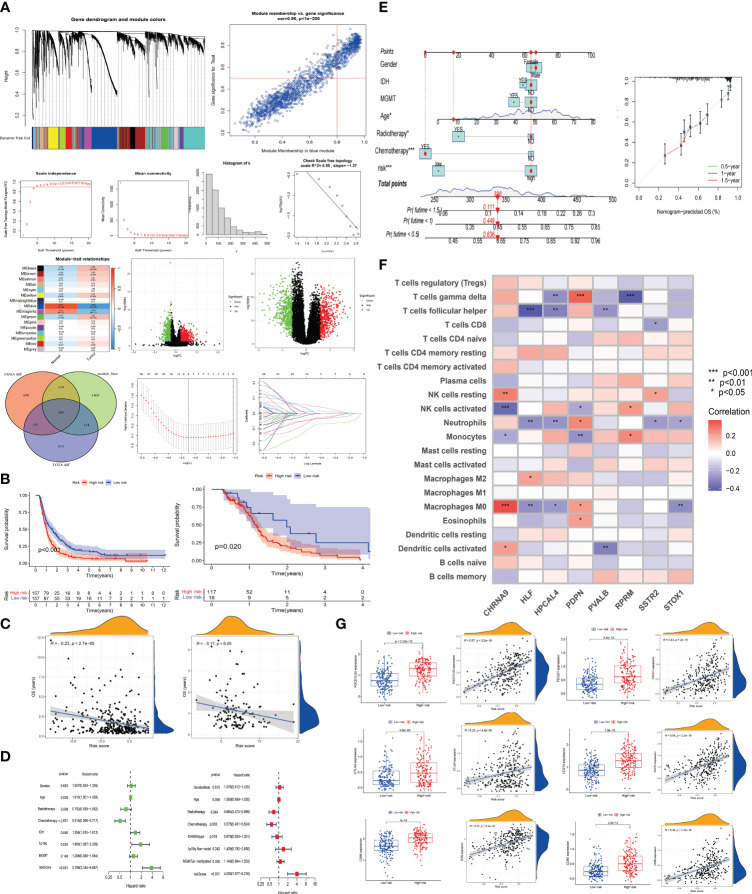
The establishment of a disulfidptosis high and low-risk model and the prediction of immunological landscape and survival risk **(A)** Utilizing WGCNA analysis, we identified a gene set most correlated with GBM occurrence. We then identified a set of genes showing significant expression differences between DRGcluster A and B subgroups in CGGA and TCGA cohorts, resulting in 105 genes. LASSO analysis was performed to establish a disulfidptosis high/low-risk prediction model. **(B)** Kaplan-Meier curves were employed to compare the survival time differences between high and low-risk groups for disulfidptosiss in CGGA and TCGA samples. **(C)** Scatter plots were used to illustrate the relationship between risk scores and survival in CGGA and TCGA samples. **(D)** Univariate and multivariate survival analyses were conducted on CGGA samples. **(E)** Nomogram and calibration plots were generated to demonstrate the predictive efficacy of survival based on various clinical indicators and risk scores. **(F)** The correlation between risk model genes and various immune cells was explored. **(G)** Expression differences of immune-related indicators in high and low-risk groups for disulfidptosiss were examined, along with their correlation with risk scores.

We utilized Weighted Gene Co-Expression Network Analysis (WGCNA) to correlate gene modules with tumor and normal symptoms characteristics of GBM patients. By comparing transcriptome data of 5 normal brain tissues from the TCGA database with 170 GBM transcriptome data, we identified a gene set in the blue module closely related to the onset of the disease (0.84, P=(7e−38)), which may play a key role in the occurrence of GBM (**Figure 4A**). In **Figure 4A**, a volcano plot displays the differential genes between DRGclusterA and B groups in CGGA and TCGA (genes with an expression difference ratio greater than 1.5 and P less than 0.05 are considered different). Visualizing these differential expression genes helps us quickly identify key genes related to disulfidptosis in GBM. TCGA data showed 1369 significant differential genes, CGGA data showed 1071, and in WGCNA’s module_blue, 1061 key genes closely related to the disease’s onset. A VENN diagram shows the common and unique genes among these three sets. The TCGA cohort is Western, and the CGGA cohort is Chinese. Intersecting these two cohorts’ data, we identified 105 common genes across different populations, which are more representative. We then entered these 105 genes into a LASSO analysis model, with the CGGA cohort as the experimental group and the TCGA cohort as the control group. After LASSO regression analysis, we obtained a gene set of 8 genes constituting the new disulfidptosis subtype in GBM. The risk score is calculated as: riskscore=CHRNA9 gene expression ×0.0459645965233746 + HLF gene expression ×-0.0755093015765612 + HPCAL4 gene expression ×0.00777741984274284 + PDPN gene expression ×0.0152203432079201 + PVALB gene expression × -0.0339601783414598 + RPRM gene expression × -0.00499112274274145 + SSTR2 gene expression × -0.0356426271018637 + STOX1 gene expression × -0.0387054324569233 (gene expression levels used FPKM data format).

KM survival analysis compared the survival time of patients in the CGGA and TCGA databases, with patients in the high-risk group showing significantly poorer survival (CGGA, p < 0.001; TCGA, p = 0.02) (**Figure 4B**). In **Figure 4C**, we conducted a correlation analysis between survival time and risk scores to assess the effectiveness of the risk model. Both CGGA and TCGA showed high consistency, with survival time significantly shortened as risk scores increased (CGGA, R = -0.23, P < 2.7e-05; TCGA, R = -0.17, P = 0.05). In **Figure 4D**, univariate and multivariate analyses were performed on the experimental group data. In the univariate analysis, age (p = 0.026), IDH status (p = 0.04), chemotherapy (p < 0.001), 1p19q status (p = 0.024), and risk score (p < 0.001) were statistically significant and had an important impact on GBM survival. In the multivariate analysis, chemotherapy (p = 0.003) and risk score (p < 0.001) were statistically significant. The HR value of the risk score was greater than 3 in both univariate and multivariate analyses, indicating that a higher risk score correlates with a higher risk of death in patients. In **Figure 4E**, factors such as gender, IDH, MGMT, age, radiotherapy, chemotherapy, and risk score were entered into a nomogram chart, where each factor was scored. By calculating these scores, a total score can be estimated, thereby predicting the patient’s survival probability at specific time points. As shown in the **Figure 4E**, when the total score is between 300-350, the patient’s 6-month survival probability is 0.636, 1-year is 0.449, and 1.5-year is 0.111. We then compared the actual observed survival probability with the nomogram-predicted survival probability through survival curves, where green, blue, and red lines represent 0.5-year, 1-year, and 1.5-year patient survival, respectively. The **Figure 4E** shows that the observed survival probability (dotted line) is close to the nomogram-predicted survival probability (solid line), indicating the nomogram’s effective prediction.

In **Figure 4F**, we displayed the correlation between various immune cell types (such as T cells, NK cells, monocytes, etc.) and the 8 risk model genes. CHRNA9 showed significant correlation with M0 macrophages, activated and dormant NK cells (p < 0.001), HLF with M0 macrophages (p < 0.01), T cells follicular helper (p < 0.001), PDPN with monocytes (p < 0.01), and HPCAL4, PDPN, RPRM all significantly correlated with T cells gamma delta (p < 0.01). CHRNA9, HLF, HPCAL4, PDPN, and STOX1 all showed significant correlation with M0 macrophages (p < 0.05). This indicates that NK cells, M0 macrophages, and certain T cell subsets (such as follicular helper) may play an important role in the development of disulfidptosis, and are highly active in regulating gene expression. In the context of disulfidptosis, these immune cells and corresponding genes have a joint response relationship. In **Figure 4G**, box plots and scatter plots showed the distribution and correlation strength and significance of immune-related indicators (CD80 (B7-1), CD86 (B7-2), CD274 (PD-L1), PDCD1 (PD-1), CTLA-4, PDCD1LG2 (PD-L2)) between high and low-risk groups. The **Figure 4G** shows that patients in the high-risk group have higher expression of immune markers (P < 0.001). At the same time, risk score and immune marker expression show a strong positive correlation (P < 0.001, R ≥ 0.23). This suggests that patients in the high-risk group may have better treatment outcomes with immunotherapy.

We conducted data analysis on both the TCGA database simultaneously, and found that their trends were roughly similar, with most P-values being statistically significant. This result validates the conclusions we obtained from the analysis of the CGGA database previously (shown in the **Supplementary Figures 3A–E**).

### Clinical application and immune analysis of disulfidptosis risk groups

**Figure 5:**
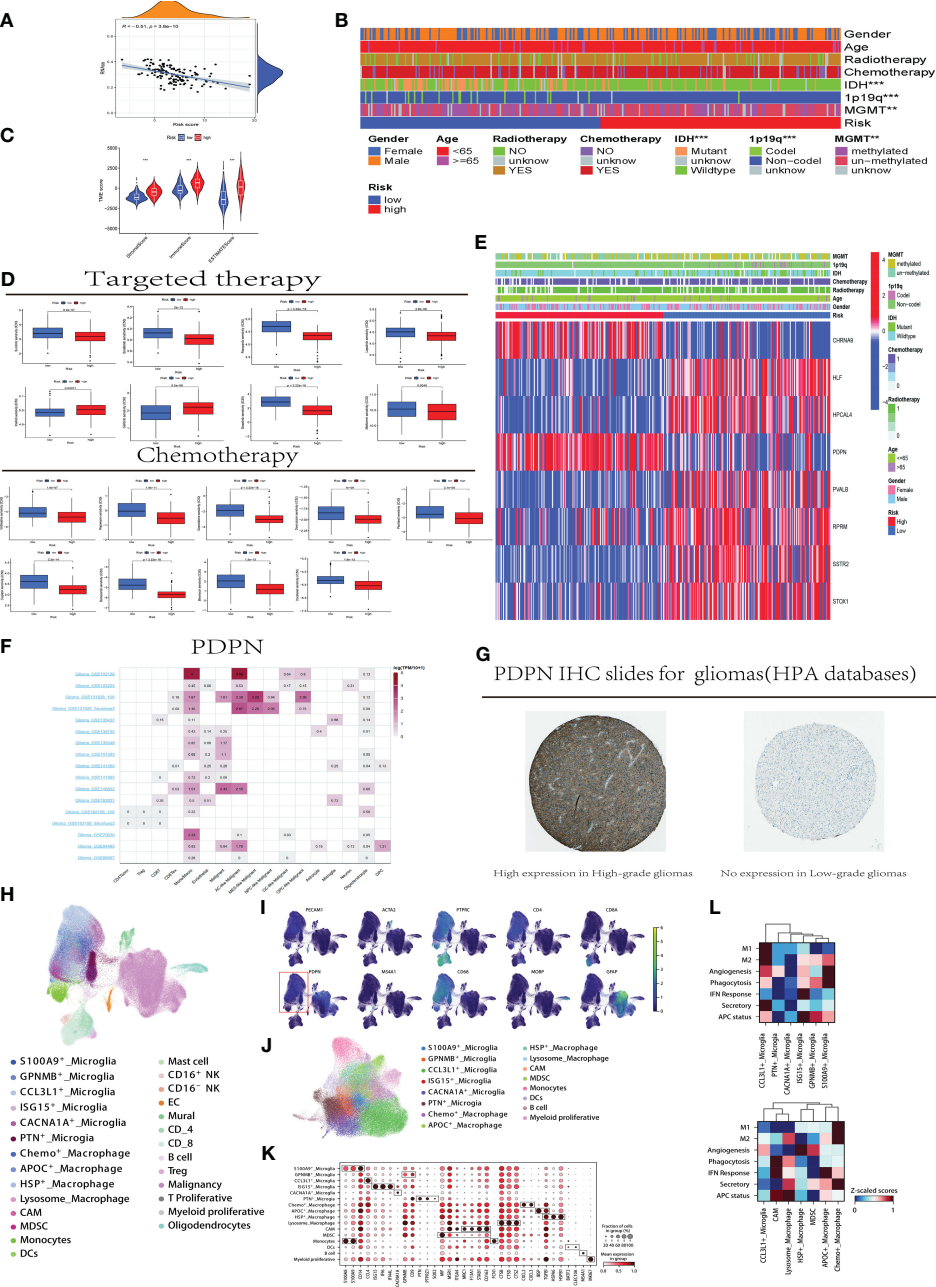
Clinical Application and Immune Analysis of Disulfidptosis Risk Groups **(A)** Correlation analysis between disulfidptosis risk scores and stem cell scores. **(B)** Distribution differences of clinical indicators in high and low-risk groups for disulfidptosis in CGGA samples. C.Analysis of immune environment differences between high and low-risk groups. **(D)** Variations in treatment responses for specific chemotherapy and targeted therapeutic drugs among patients in high and low-risk groups. **(E)** Association analysis between the expression of risk model genes in high and low-risk groups and clinical indicators. **(F)** Utilizing the TISCH website to analyze the differential gene expression of PDPN in different immune cells and GBM tumor cell subgroups. **(G)** Downloading immunohistochemistry (IHC) slides from the HPA database to demonstrate the differential expression of PDPN protein in high-grade (left) and low-grade (right) samples, with the left slide indicating high expression and the right slide showing no expression. **(H)** Reclassify the collected tumor single cells from all patients. **(I)** Distribution of the PDPN gene in single-cell subpopulations within tumors and the microenvironment. **(J)** Single cell myeloid subpopulations. **(K)** Further defining the functional status of myeloid cells using cell markers. L. Scoring of cellular functional processes in macrophage and microglia cells. CCL3L1, C-C Motif Chemokine Ligand 3 Like 1; PTN, Pleiotrophin; CACNA1A, Calcium Voltage-Gated Channel Subunit Alpha1 A; ISG15, Interferon-Stimulated Gene 15; GPNMB, Glycoprotein Nmb; S100A9, S100 Calcium Binding Protein A9; HSP, Heat Shock Protein; MDSC, Myeloid-Derived Suppressor Cell; APOC, Apolipoprotein C; M1/M2, M1 macrophage/M2 macrophage; APC, Antigen Presenting Cell; IFN, Interferon; TAM, Tumor-associated macrophages; MDSC, Monocytic-derived suppressor cells; MIF, Macrophage migration inhibitory factor.

In **Figure 5A**, the scatter plot demonstrating the correlation between disulfidptosis risk score and stem cell score reveals that higher risk scores are associated with lower stem cell scores, indicating a significant correlation (P=3.8e-10, R=-0.51). **Figure 5B** assesses the distribution differences in gender, age, treatment (radiotherapy, chemotherapy), and genetic markers (IDH, 1p/19q, MGMT) among populations in high and low disulfidptosis risk groups. It was found that patients with IDH mutation, 1p19q co-deletion, and MGMT methylation are more likely to be in the high-risk group for disulfidptosis (p < 0.001), suggesting that these biomarkers may be related to disulfidptosis occurrence and could serve as predictive factors for disulfidptosis risk, guiding personalized treatment. In **Figure 5C**, the violin plots compare the immune environment scores between high and low risk groups for disulfidptosis, showing significantly higher scores in the high-risk group (p < 0.001), which may imply a correlation between changes in the immune environment and disulfidptosis risk.

**Figure 5D** compares the responses to common chemotherapy and targeted treatments in high and low disulfidptosis risk groups. Lapatinib, a selective inhibitor of the HER2 target, has shown inhibitory effects on HER2-positive GBM cells in some early *in vitro* and animal studies, but this does not necessarily translate into significant clinical treatment effects. The **Figure 5D** reveals that patients in the disulfidptosis risk group have a higher IC50 value for Lapatinib compared to those in the high-risk group (p < 0.001), indicating that patients in the high disulfidptosis risk group may have a better response to Lapatinib, and the disulfidptosis process might affect the treatment response to Lapatinib. Cisplatin, commonly used in second-line and subsequent treatments for GBM, is shown to have significantly better responsiveness in the high-risk group. Additionally, an interesting phenomenon was observed in our study: patients in high and low disulfidptosis risk groups show significant differences in response to metformin treatment (p < 0.01). In **Figure 5E**, the heatmap combining the expression of the eight genes constituting the disulfidptosis risk model with clinical indicators shows significant differential expression of the eight model genes between high and low-risk groups. **Figure 5F**, utilizing TISCH website data analysis, display the expression of the PDPN gene in different immune cells. Higher expression is observed in glioblastoma cells, monocytes, macrophages, and T cell subgroups (such as CD8Tex), while lower expression is noted in low-grade gliomas and other immune cells in the immune environment, suggesting that high expression of PDPN may be related to the development, invasion, and immune response of glioblastoma. Immunohistochemistry (IHC) images downloaded from the HPA database show higher expression of PDPN protein in high-grade gliomas compared to low-grade gliomas (**Figure 5G**).

Then the 42 samples from 16 GBM patients generated 227,584 single-cell transcriptomes after the quality control. Data was normalized and merged using Scanpy, and visualized via uniform manifold approximation and projection (UMAP) (**Figure 5H**). For Initial identification of disease type, based on expression levels of canonical marker genes (**Figure 5I**, and genes included: PTPRC for immune cell; CD4, CD8A and MS4A1 for lymphocytes; CD68 for myeloid cell; MPBP for oligodendrocyte; GFAP for malignancy; PECAM1 for endothelial cell; ATCA2 for mural cell). As shown in the **Figure 5I**, the malignancy and myeloid cell mainly expressed the PDPN.

In the myeloid cell, we used the canonical marker (TEME19 and P2RY12 for microglia) to distinguish microglia from the blood-derived monocytes/macrophages. And then, the microglia were dissected into 6 group (**Figures 5J, K**). A subpopulation unregulated(S100A9+_Microglia: S100A8 and S100A9), an immune regulatory microglia(GPNMB+_Microglia: GPNMB and CD9), formerly reported as TAM promoting cancer metastasis and stemness in macrophage (14), a chemokinase microglia subtype(CCL3L1+_Microglia:CCL4 and CCL3L1 etc.), an ISG microglia cluster(ISG15+_Microglia:ISG15,IFI6 and IFI44L), a precursor-like microglia(PTN+_ Microglia: PTN, PTPRZ1 and SOX2), secreting PTN to significantly promote tumor invasion (15) and a subgroup(CACNA1A+_Microglia) highly expressed CACNA1A with unclear function.

As for the macrophages/monocytes, 7 subtypes were identified (**Figure 5L**), including Chemo+_Macrophage (higher expression of CXCL2 and CXCL3), APOC+_Macrophage(higher expression of APOC, IBSP and TGFBI (16)), HSP+_Macrophage characterized by HSPA6 and HSPB1, Lysosome_Macrophage upregulating CTSB, CTSD and CTSZ), CNS-associated Macrophage identified by MRC1,F13A1 and STAB1 (17), monocytes derived suppressor cell(MDSC) determined by higher expression of MIF and lower expression of mature signature and monocytes.

## Discussion

Glioblastoma multiforme (GBM) is considered the most malignant and deadly intracranial tumor, posing significant therapeutic challenges due to limited treatment options (18). Given the pronounced heterogeneity in the prognosis of GBM patients, subtype classification is crucial for precision medicine (19). The standard first-line chemotherapy regimen for GBM typically includes temozolomide (TMZ) and antimetabolites such as temsirolimus (20). However, treatment responses for GBM are inconsistent, and resistance to these drugs is gradually becoming a challenge. Researchers are actively seeking new chemotherapeutic agents to enhance treatment efficacy. Clinical trials are also exploring novel drug combinations and therapies. Some GBM patients undergo immune checkpoint inhibitor therapy, such as PD-1/PD-L1 blockade, to bolster the immune system’s attack on tumors. Nevertheless, GBM often evades immune surveillance, limiting the effectiveness of immunotherapy. Personalized immunotherapy vaccines, utilizing the patient’s own tumor tissue or cells, represent an emerging treatment approach to stimulate the immune system’s response against tumors (21). Some GBM patients exhibit overexpression of epidermal growth factor receptor (EGFR) (22). Drugs like lapatinib are designed to inhibit EGFR, but the clinical outcomes in studies remain inconsistent. Pathway inhibitors such as everolimus targeting the PI3K/AKT/mTOR pathway are also employed in an attempt to halt glioblastoma multiforme growth. Despite some progress, GBM treatment remains a formidable challenge. Researchers continuously seek more effective treatment modalities, including gene therapy, immunotherapeutic approaches (23), and other innovative methods, to improve patient survival and quality of life. Clinical trials and personalized treatment strategies are crucial for better understanding and addressing the complexity of GBM (23).

Recently discovered disulfidptosis, a novel form of cell death, is closely associated with aberrant tumor glucose metabolism, particularly in tumors with elevated SLC7A11 expression. Previous studies have indicated that gliomas exhibit high SLC7A11 expression, heightened metabolism, and glucose deficiency. Consequently, we delved into the role of disulfidptosis in glioblastoma multiforme. Disulfidptosis represents a regulated form of cell death controlled by proteins such as SLC7A11 and NCKAP1, along with drugs possessing disulfide reductase activity. Under glucose-deficient conditions, cells with elevated SLC7A11 levels undergo abnormal accumulation of disulfide bonds, such as cysteine. This accumulation triggers disulfide stress, elevating the disulfide content in the cell’s cytoskeleton, causing structural damage, and ultimately leading to cell death. Recent research highlights the pivotal role of SLC7A11-mediated redox status in various aspects of tumor growth, including multidrug resistance, tumor-associated disulfidptosis, and iron-dependent cell death. Notably, regulatory proteins such as SLC7A11 and NCKAP1, critical in disulfidptosis, also play essential roles in glioma onset and progression. This suggests that disulfidptosis may indeed be a significant contributor to the pathogenesis of GBM.

We propose a novel classification of GBM based on the characteristics of tumor disulfidptosis. DRGclusterA group exhibited higher expression levels of immune checkpoint molecules, including CD80 (24), CD86 (25), CTLA4 (26), PDCD1, PDCD1LG2, and CD274 (27), compared to the DRGclusterB group, with statistically significant differences in the expression of these checkpoints between the two clusters. PD-1 ligand 2 (PDCD1LG2), also known as PD-L2, and programmed cell death 1 ligand 1 (CD274 or PD-L1) were among the identified checkpoint molecules. PD-L2 is primarily expressed on antigen-presenting cells such as dendritic cells and macrophages, while PD-L1 is expressed on various cell surfaces, including cancer cells and antigen-presenting cells, playing a crucial role in immune regulation. CD80 and CD86 are molecules present on the surface of antigen-presenting cells, contributing to T cell activation through interaction with the CD28 molecule. CTLA-4, located on T cell surfaces, acts as an inhibitory receptor, suppressing T cell activation and immune responses. Programmed cell death 1 (PDCD1 or PD-1) is a membrane receptor expressed on activated T cells, B cells, and macrophages, modulating immune responses by inhibiting T cell activation. The high expression of immune checkpoint molecules in the DRGclusterA group suggests potential sensitivity to immune therapy. Immune checkpoint inhibitors, such as anti-PD-1, PD-L1, and CTLA-4 antibodies, have achieved significant success in the treatment of various cancers. These therapeutic approaches may be more effective for patients in the DRGclusterA group. The low expression of immune checkpoint molecules in the DRGclusterB group may involve the activation of immune escape mechanisms. In-depth understanding of this mechanism can provide clues for developing new treatment strategies to enhance the immune response in these patients.

Simultaneously, we categorized GBM patients into two immune subtypes: High Immunity (Immunity_H) and Low Immunity (Immunity_L). The DRGclusterA group displayed higher immune, stromal, and ESTIMATE scores, while the DRGclusterB group exhibited higher tumor purity. Unsupervised hierarchical clustering classified CGGA, TCGA, and TianTan patients into these two subtypes, with DRGclusterA predominantly corresponding to the high immune group and DRGclusterB to the low immune group. In the high immune group (DRGclusterA), a significant correlation was observed with T cell subtypes, NK cells, plasma cells, M0 macrophages, and monocytes. The DRGclusterA group displayed higher immune and stromal cell abundances, suggesting that this population may be more suitable for immune checkpoint inhibitor drugs, potentially facilitating immune system activation. However, further clinical and foundational research is needed to validate and deepen our understanding of these findings.

Furthermore, using pRRophetic (28), we predicted the sensitivity of DRGclusterA and B patients to specific drugs. Based on the provided information, DRGcluster A exhibits higher activity in multiple pathways, including various cell signaling, metabolic pathways, and cell cycle regulation. Here’s a further analysis of the correlation between the increased activity in these pathways and the enhanced drug sensitivity: (1).Increased Activity in Cell Signaling Pathways: DRGcluster A shows higher activity in several cell signaling pathways relevant to cancer, such as EGFR, VEGF, JAK/STAT, P53, MAPK, and TGF-beta. These pathways play crucial roles in proliferation, survival, and apoptosis of tumor cells. Therefore, DRGcluster A might be more sensitive to drugs targeting these pathways, such as Dasatinib, Sunitinib, Lapatinib, Imatinib, etc., which could effectively inhibit their activity and impact the growth and metastasis of glioblastoma.(2). Increased Activity in Cell Cycle Regulation Pathways: DRGcluster A also demonstrates higher activity in pathways related to cell cycle regulation, apoptosis, DNA repair, etc. Aberrant activity in these pathways may lead to dysregulation of the cell cycle and increased apoptosis, making DRGcluster A more sensitive to drugs affecting the cell cycle and apoptosis, such as Cisplatin, Docetaxel, Paclitaxel, etc. (3). Modulation of Metabolic Pathways:DRGcluster A shows increased activity in various metabolic pathways, including glucose metabolism, amino acid metabolism, lipid metabolism, etc. This modulation could affect drug metabolism and intracellular environments, thereby influencing the mode of action and efficacy of drugs. For drugs targeting metabolic pathways, such as Metformin, DRGcluster A may exhibit higher sensitivity. (4). Increased Activity in Cell Adhesion and Migration Pathways: DRGcluster A demonstrates higher activity in pathways related to cell adhesion, leukocyte transendothelial migration, etc., which are associated with tumor metastasis and infiltration. Therefore, drugs targeting these pathways, such as Sorafenib, may exhibit higher sensitivity in DRGcluster A, which could inhibit tumor dissemination and metastasis effectively. In summary, the heightened activity of DRGcluster A in these pathways may lead to increased sensitivity to drugs that target these pathways. This further supports the explanation for the enhanced sensitivity to chemotherapy drugs like Cisplatin, Docetaxel, Paclitaxel, as well as targeted therapy drugs like Dasatinib, Sunitinib, Metformin, etc

Subsequently, we employed LASSO machine (29) learning to establish a disulfidptosis risk model, comparing the survival time of GBM patients using Kaplan-Meier survival analysis. Patients in the high-risk disulfidptosis group exhibited significantly poorer survival outcomes. The high-risk group demonstrated higher expression levels of immune biomarkers (P < 0.001). Strong positive correlations were observed between risk scores and immune biomarker expression (P < 0.001, R ≥ 0.23), suggesting that patients in the high-risk group may benefit more from immune therapy. Stem cell scores typically reflect the presence and relative quantity of stem cells in a sample (30). Stem cells play crucial roles in various biological processes, including tissue regeneration, development, and the onset and progression of GBM. Scatter plots depicting the correlation between disulfidptosis risk scores and stem cell scores showed a significant negative correlation (P = 3.8e-10, R = -0.51). This indicates that higher disulfidptosis risk scores are associated with lower stem cell scores, suggesting that biological processes occurring in patients at high risk for disulfidptosis may lead to a reduction in the quantity or activity of stem cells. Modulating stem cell activity could potentially reduce the risk of disulfidptosis. Decreased stem cell scores under disulfidptosis conditions may serve as an early biological indicator for predicting the occurrence of disulfidptosis.

We also compared the common chemotherapy and targeted treatment responses in the high and low disulfidptosis risk groups. Lapatinib, a selective inhibitor of the HER2 target, has shown inhibitory effects on HER2-positive GBM cells in some early *in vitro* and animal studies. However, this efficacy has not necessarily translated into significant clinical outcomes.Patients in the high-risk disulfidptosis group may have a better response to Lapatinib, and the process of disulfidptosis may influence the therapeutic response to Lapatinib (31), Cisplatin, commonly used in second-line and subsequent treatments for GBM, showed a significantly better response in the high-risk group compared to the low-risk group, suggesting that patients with lower disulfidptosis risk may need to consider alternative treatment strategies or adopt combination therapy. In our study, an interesting phenomenon was also observed regarding the treatment response to metformin, with significant differences between the high and low disulfidptosis risk groups. Previous research has suggested that metformin could be a potential anti-tumor drug, affecting tumor cells through various mechanisms, including inhibiting cell proliferation, regulating energy metabolism, and influencing tumor stem cells. The differential response to metformin provides insights into further studying the anti-tumor mechanisms of metformin in specific subgroups of GBM. Metformin could be considered as a supplementary treatment for patients in the high-risk disulfidptosis group or for combination therapy, potentially enhancing overall treatment efficacy.

In our risk model, PDPN (Podoplanin), a gene encoding the epidermal growth factor receptor (EGFR) ligand, was identified as a predictive factor (32). Podoplanin is commonly associated with tumor invasion and metastasis. In the immune system, PDPN is also related to immune regulation and inflammatory processes (33). The single-cell analysis data revealed elevated expression of the PDPN gene in different immune cells, such as GBM cells, monocytes, and macrophages. Higher PDPN protein expression was observed in high-grade gliomas compared to low-grade gliomas, as shown in immunohistochemistry images downloaded from the HPA database. This suggests that high expression of PDPN may be associated with the development, invasion, and immune response of glioblastoma multiforme, indicating its potential relevance to the invasive nature, recurrence, and poor prognosis of tumors.

## Conclusions

The results of this study underscore the significant association between GBM patients and disulfidptosis. The establishment of our DRGcluster grouping and disulfidptosis risk model demonstrates its effectiveness in evaluating GBM prognosis, cellular functions, tumor microenvironment, and somatic cell mutations. These findings provide hope for tailoring GBM treatment strategies and selecting appropriate medications.Future research could delve into exploring novel therapeutic targets stemming from the identified cellular mechanisms and somatic mutations. Moreover, the application of our risk model in clinical settings may facilitate personalized treatment approaches for GBM patients, potentially improving their overall prognosis and quality of life.

## Data availability statement

The data presented in the study are deposited in the NCBI GEO repository, accession number GSE 252709.

## Ethics statement

The studies involving humans were approved by the Ethics Committee of Tiantan Hospital, Capital Medical University. The studies were conducted in accordance with the local legislation and institutional requirements. Written informed consent for participation in this study was provided by the participants’ legal guardians/next of kin.

## Author contributions

XY: Data curation, Investigation, Writing – original draft, Writing – review & editing. ZC: Investigation, Software, Writing – review & editing. CW: Formal analysis, Investigation, Methodology, Visualization, Writing – review & editing. CJ: Conceptualization, Data curation, Investigation, Methodology, Writing – original draft. JL: Funding acquisition, Investigation, Project administration, Writing – original draft. FC: Investigation, Methodology, Project administration, Writing – review & editing. WL: Data curation, Formal analysis, Funding acquisition, Investigation, Methodology, Project administration, Supervision, Validation, Writing – review & editing.

## Supplementary material

SUPPLEMENTARY FIGURE 1 The flow chart of the article.

SUPPLEMENTARY FIGURE 2 The association between the DRGclusters and the immune microenvironment pattern S2A. Differential expression of HLA family genes in patients stratified by high and low immune groups in the TCGA cohort. S2B.Box plots depicting differences in immune cell infiltration among patients with different immune subtypes in the TCGA cohort. S2C. Scatter plots comparing the expression levels of multiple immune-related genes in different DRGcluster subtypes in the TCGA cohort. S2D. Differential expression of HLA family genes in patients stratified by DRGcluster A and B groups in the Tiantan cohort. S2E. Box plots depicting differences in immune cell infiltration among patients with different DRGcluster subtypes in the Tiantan cohort.S2F. Scatter plots comparing the expression levels of multiple immune-related genes in different DRGcluster subtypes in the Tiantan cohort.

SUPPLEMENTARY FIGURE 3 The establishment of a disulfidptosis high and low-risk model and the prediction of immunological landscape and survival risk S3A. calibration plots were generated to demonstrate the predictive efficacy of survival based on various clinical indicators and risk scores in the TCGA cohort. S3B.Evaluating the predictive efficiency of models using ROC curves in the CGGA cohort. S3C.Evaluating the predictive efficiency of models using ROC curves in the TGGA cohort. S3D.Nomogram was generated to demonstrate the predictive efficacy of survival based on various clinical indicators and risk scores. S2E. Scatter plots comparing the expression levels of multiple immune-related genes in different disulfidptosis risk subtypes in the TCGA cohort.
