# Supplementary figures and images for: Integrated multiomic analysis reveals disulfidptosis subtypes in glioblastoma: implications for immunotherapy, targeted therapy, and chemotherapy
Source: Front Immunol. 2024 Feb 26;15:1362543. doi: 10.3389/fimmu.2024.1362543 (PMC10950096; doi:10.3389/fimmu.2024.1362543)

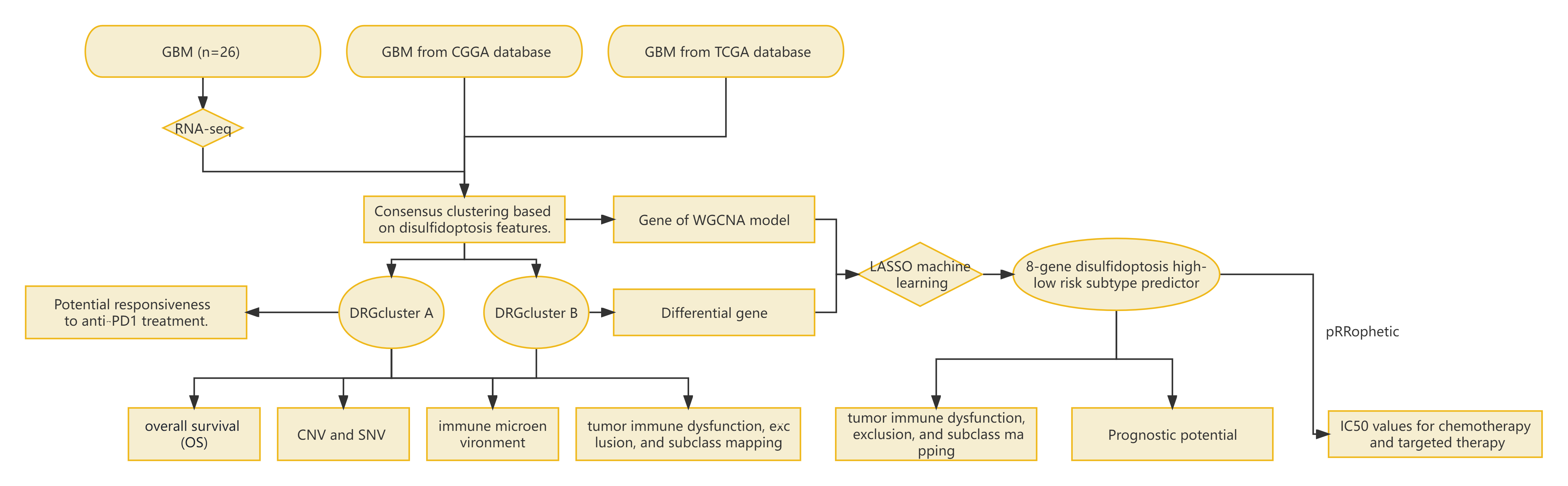

Supplement: SUPPLEMENTARY FIGURE 1 — The flow chart of the article. [file Image_1.png]

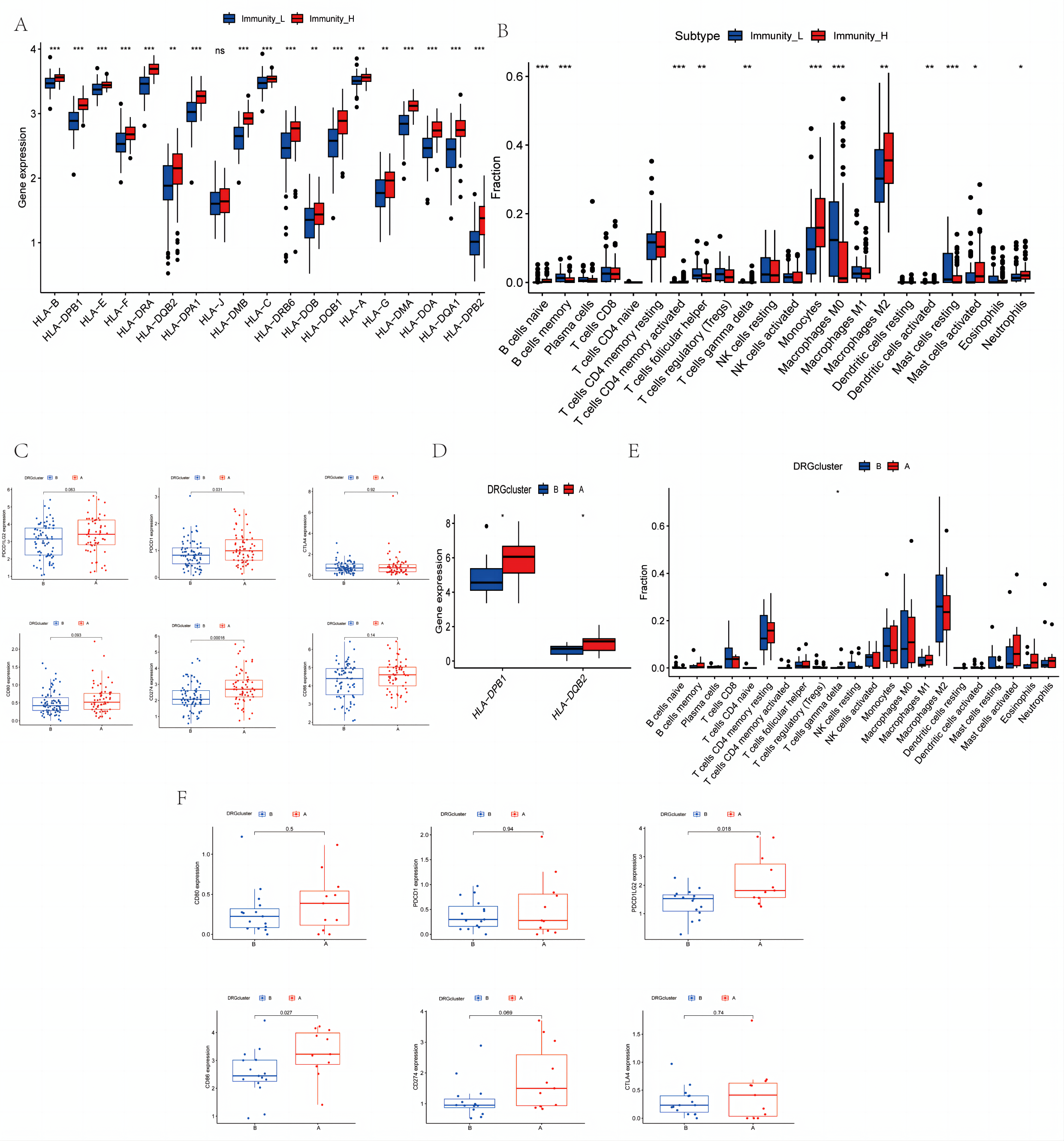

Supplement: SUPPLEMENTARY FIGURE 2 — The association between the DRGclusters and the immune microenvironment pattern S2A. Differential expression of HLA family genes in patients stratified by high and low immune groups in the TCGA cohort. S2B.Box plots depicting differences in immune cell infiltration among patients with different immune subtypes in the TCGA cohort. S2C. Scatter plots comparing the expression levels of multiple immune-related genes in different DRGcluster subtypes in the TCGA cohort. S2D. Differential expression of HLA family genes in patients stratified by DRGcluster A and B groups in the Tiantan cohort. S2E. Box plots depicting differences in immune cell infiltration among patients with different DRGcluster subtypes in the Tiantan cohort.S2F. Scatter plots comparing the expression levels of multiple immune-related genes in different DRGcluster subtypes in the Tiantan cohort. [file Image_2.png]

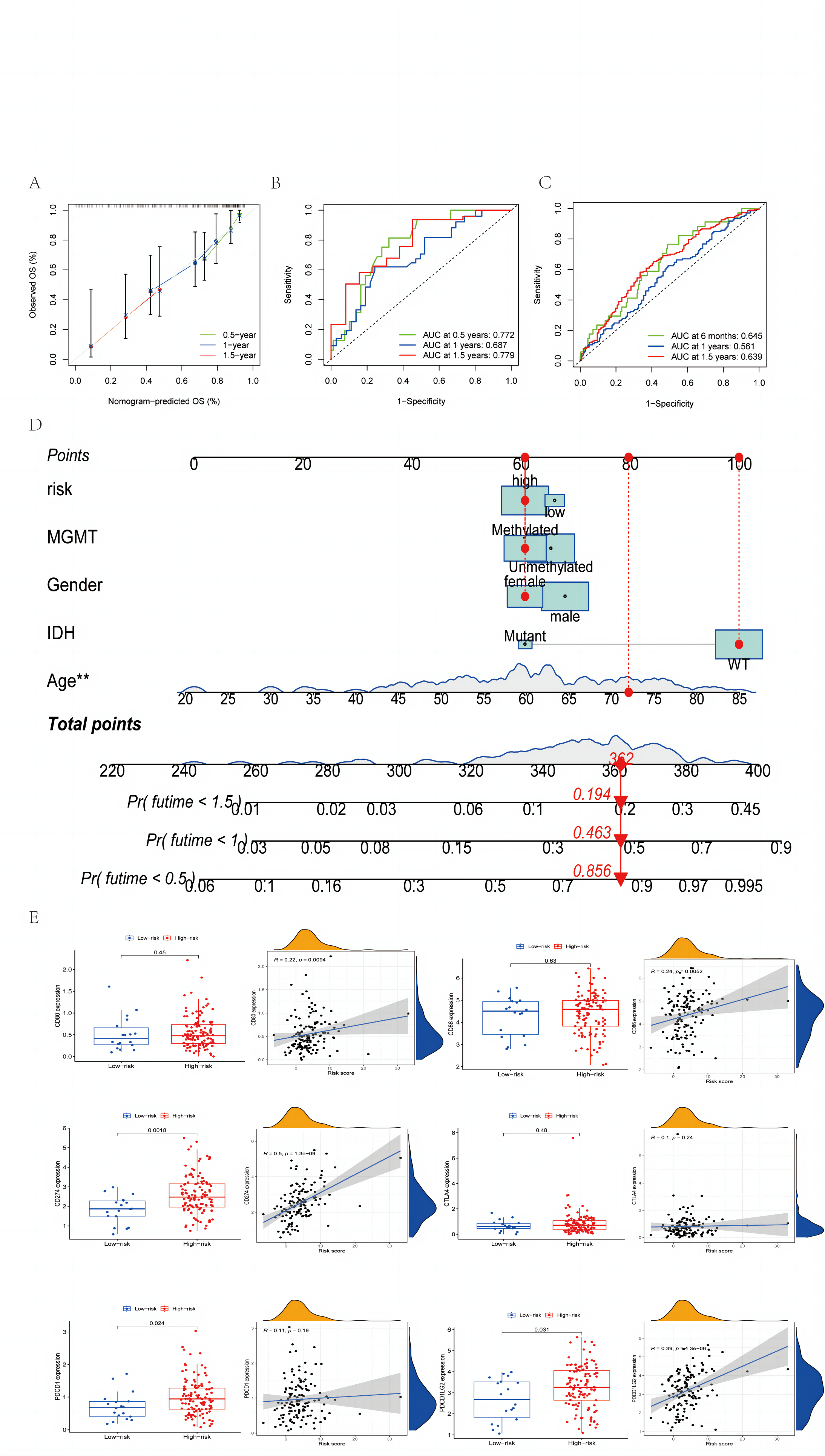

Supplement: SUPPLEMENTARY FIGURE 3 — The establishment of a disulfidptosis high and low-risk model and the prediction of immunological landscape and survival risk S3A. calibration plots were generated to demonstrate the predictive efficacy of survival based on various clinical indicators and risk scores in the TCGA cohort. S3B.Evaluating the predictive efficiency of models using ROC curves in the CGGA cohort. S3C.Evaluating the predictive efficiency of models using ROC curves in the TGGA cohort. S3D.Nomogram was generated to demonstrate the predictive efficacy of survival based on various clinical indicators and risk scores. S2E. Scatter plots comparing the expression levels of multiple immune-related genes in different disulfidptosis risk subtypes in the TCGA cohort. [file Image_3.png]

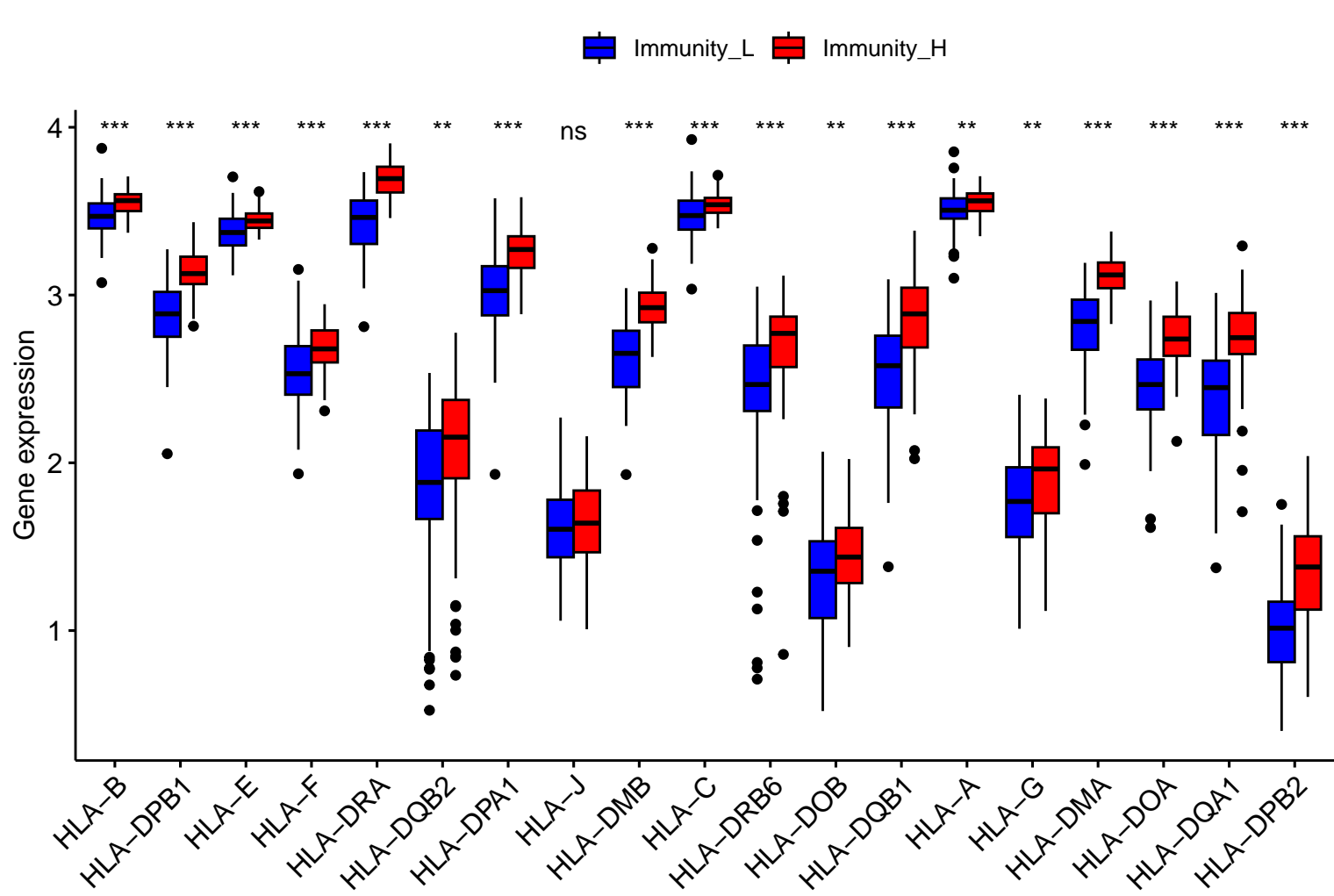

Supplement: Supplementary file 5 [file DataSheet_1.pdf]

Subtype Immunity\_L Immunity\_H

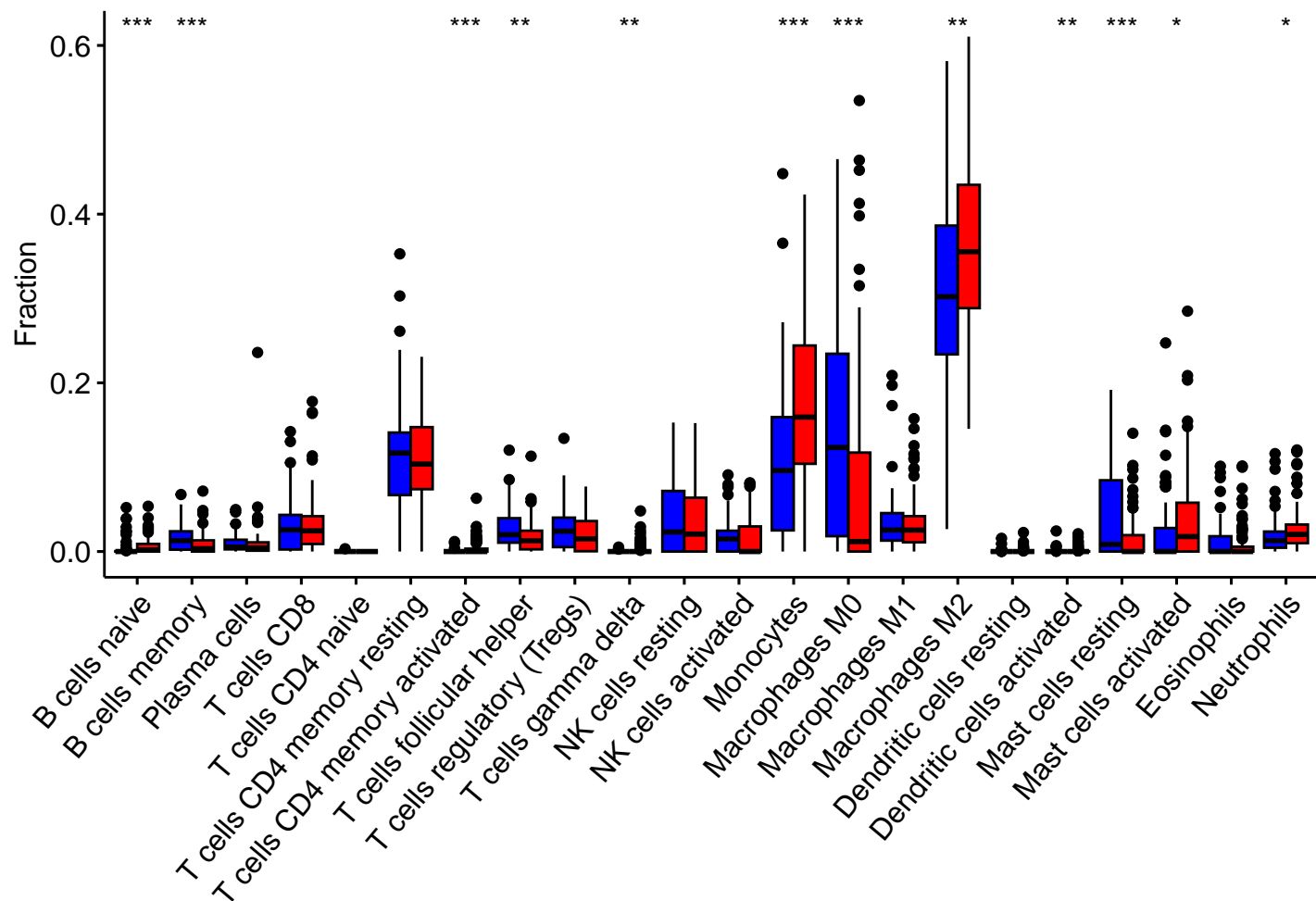

Supplement: Supplementary file 6 [file DataSheet_2.pdf]

DRGcluster B A

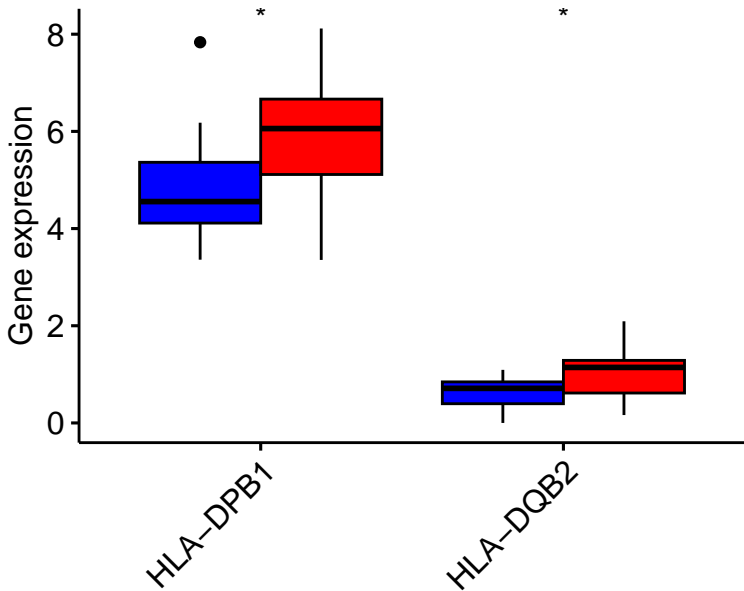

Supplement: Supplementary file 7 [file DataSheet_3.pdf]

DRGcluster B A

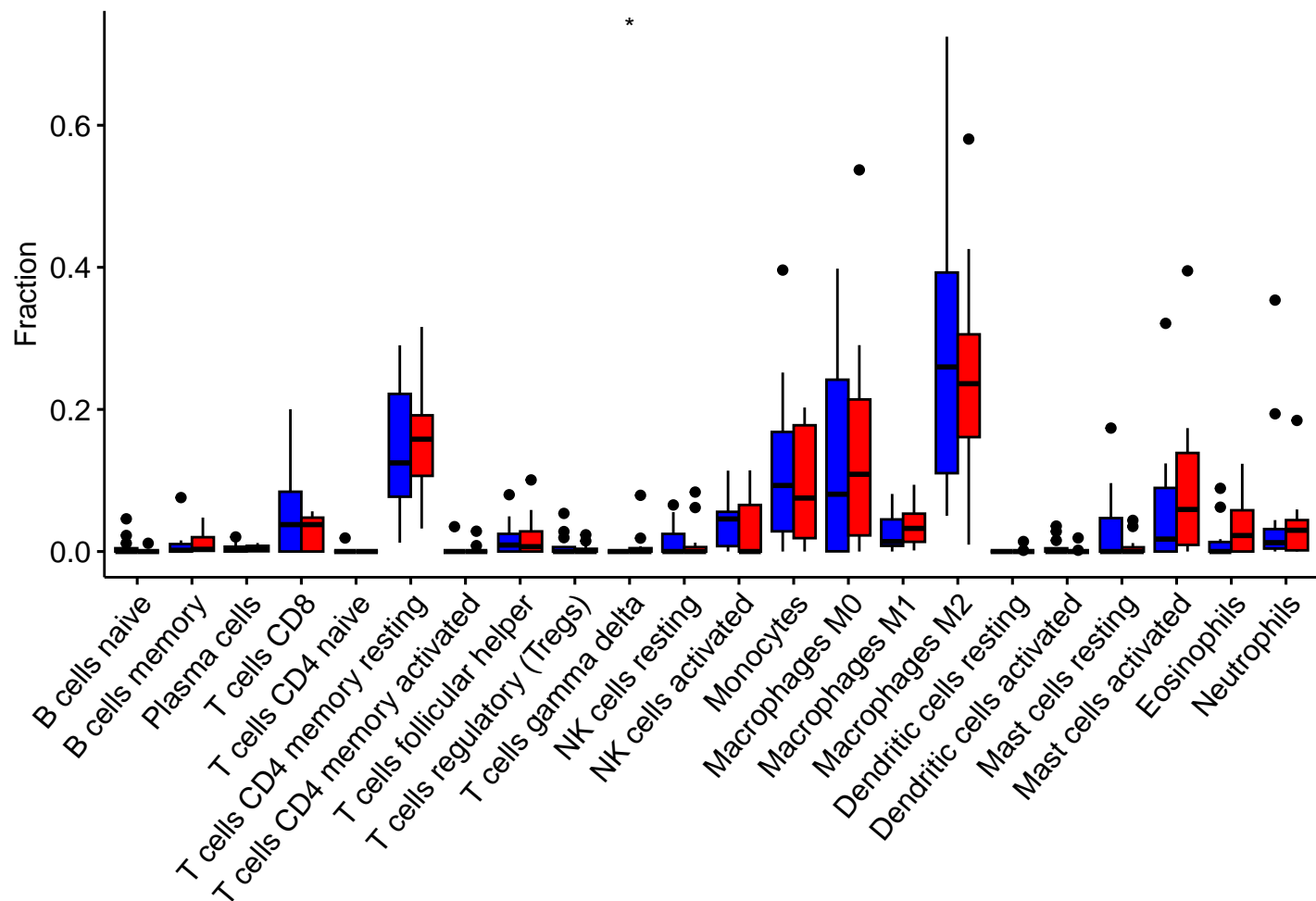

Supplement: Supplementary file 8 [file DataSheet_4.pdf]

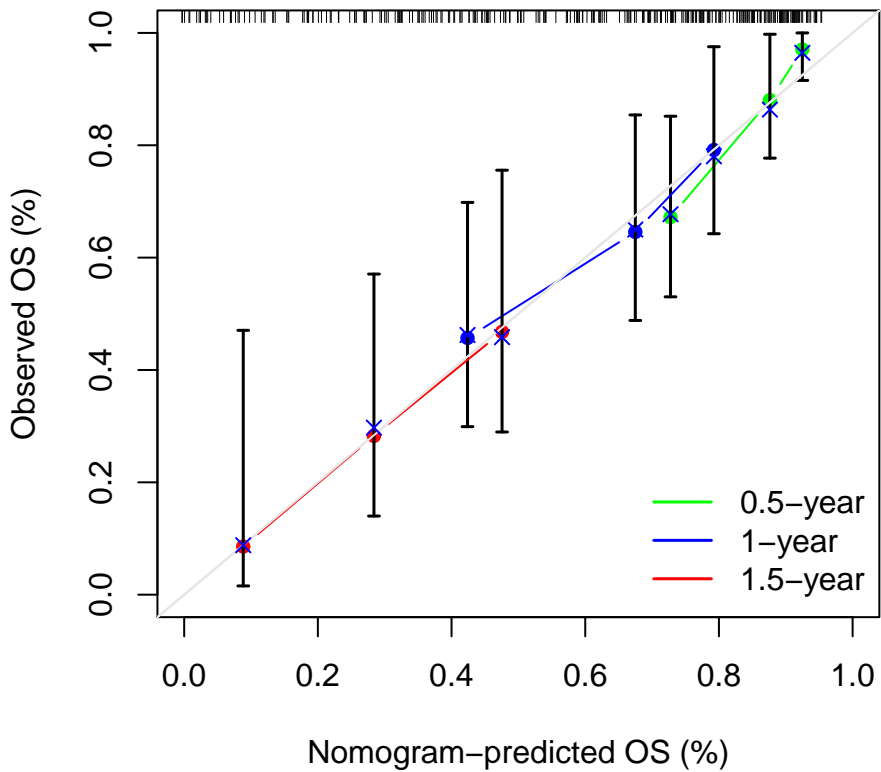

Supplement: Supplementary file 9 [file DataSheet_5.pdf]

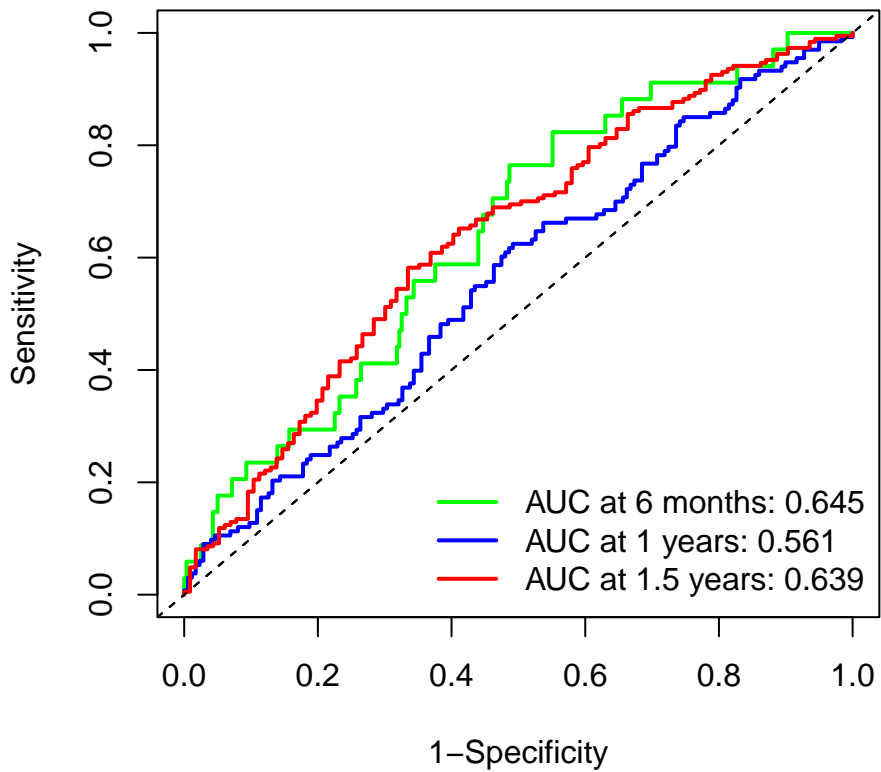

Supplement: Supplementary file 10 [file DataSheet_6.pdf]

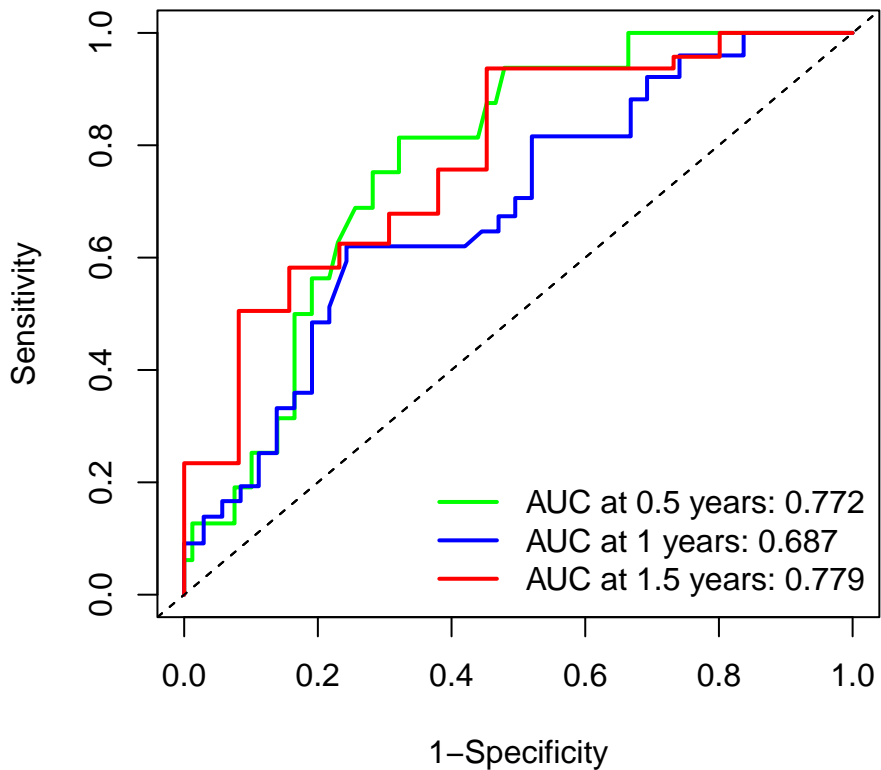

Supplement: Supplementary file 11 [file DataSheet_7.pdf]

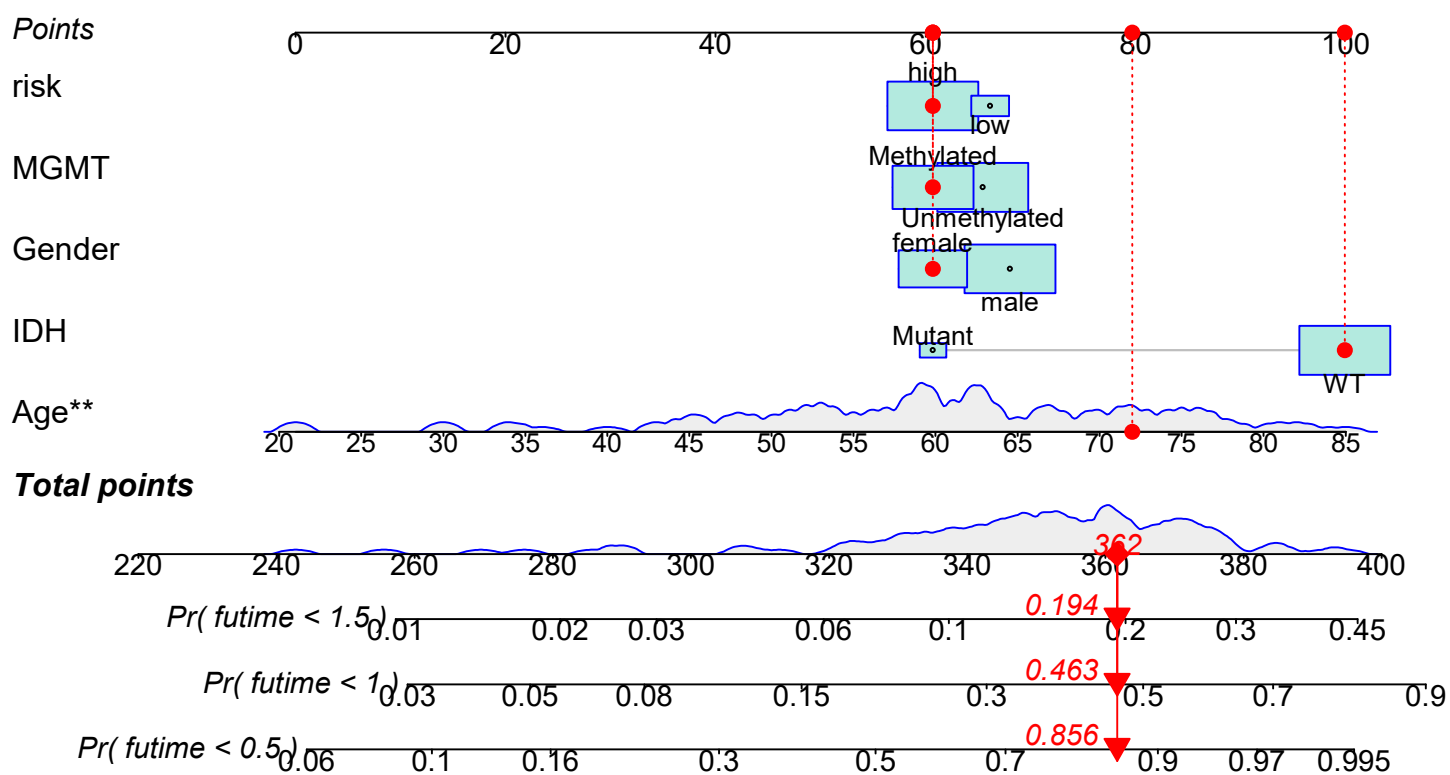

Supplement: Supplementary file 12 [file DataSheet_8.pdf]
